# Did the exposure of coacervate droplets to rain make them the first stable protocells?

**DOI:** 10.1126/sciadv.adn9657

**Published:** 2024-08-21

**Authors:** Aman Agrawal, Aleksandar Radakovic, Anusha Vonteddu, Syed Rizvi, Vivian N. Huynh, Jack F. Douglas, Matthew V. Tirrell, Alamgir Karim, Jack W. Szostak

**Affiliations:** ^1^ William A. Brookshire Department of Chemical & Biomolecular Engineering, University of Houston, Houston, TX 77204, USA.; ^2^ Pritzker School of Molecular Engineering, The University of Chicago, Chicago, IL 60637, USA.; ^3^ Howard Hughes Medical Institute, Department of Chemistry, The University of Chicago, Chicago, IL 60637, USA.; ^4^ Materials Science and Engineering Program, University of Houston, Houston, TX 77204, USA.; ^5^ Materials Science and Engineering Division, National Institute of Standards and Technology, Gaithersburg, MD 20899, USA.; ^6^ Argonne National Laboratory, Lemont, IL, 60439 USA.

## Abstract

Membraneless coacervate microdroplets have long been proposed as model protocells as they can grow, divide, and concentrate RNA by natural partitioning. However, the rapid exchange of RNA between these compartments, along with their rapid fusion, both within minutes, means that individual droplets would be unable to maintain their separate genetic identities. Hence, Darwinian evolution would not be possible, and the population would be vulnerable to collapse due to the rapid spread of parasitic RNAs. In this study, we show that distilled water, mimicking rain/freshwater, leads to the formation of electrostatic crosslinks on the interface of coacervate droplets that not only suppress droplet fusion indefinitely but also allow the spatiotemporal compartmentalization of RNA on a timescale of days depending on the length and structure of RNA. We suggest that these nonfusing membraneless droplets could potentially act as protocells with the capacity to evolve compartmentalized ribozymes in prebiotic environments.

## Teaser

Electrostatic crosslinks on membraneless protocells in distilled water stabilize them against fusion and rapid RNA exchange.

## INTRODUCTION

One of the most fascinating questions in science is how life originated on Earth. Specifically, how did nonliving matter transform into living cells that can perform complex functions such as replication, metabolism, and evolution? One of the hypotheses for the origin of life is that local prebiotic environments on the early Earth contained many of the now-recognized molecular building blocks of living cells, thereby allowing the self-assembly of minimalistic model cell structures or protocells (*1*, *2*). Protocells are crucial for the emergence of life because they provide compartmentalization for concentration, organization, and replication of essential biomolecules, the most important being ribonucleic acids or RNAs (*3*, *4*). RNA is considered a key molecule in the prebiotic world and the origin of life because of its dual functions of storing genetic information and catalyzing chemical reactions (*5*). According to the RNA-world hypothesis, RNA was the first biopolymer to have emerged from simpler organic molecules and formed self-replicating systems that could evolve and diversify (*6*). Another prevalent hypothesis in the origin of life community is that the compartmentalization of RNA within protocells could have arisen naturally from the self-assembly of vesicles of amphiphilic molecules, such as lipids or fatty acids, that enclose an aqueous core in which reactions critical to metabolism and reproduction can occur (*7*, *8*). While these protocells resemble modern cells, they lack what is now a highly evolved membrane structure that supports molecular trafficking features. Although lipid or fatty acid membranes can provide selectivity and permeability to the encapsulated region, only small molecules, such as nucleotides and their dimers or trimers, can permeate through the bilayer (*9*, *10*). As the size of RNA increases, the protocells would have required specific transport proteins to facilitate their passage across the cell membrane (*11*). The existence of intricate membrane proteins at the origin of compartmentalization seems highly improbable.

A popular alternative hypothesis is that primordial protocells could have arisen from the formation of coacervates in which there is no lipid membrane. This idea was first proposed by Oparin in his pioneering work on the origin of life in the 1930s, following the description of coacervates by Bungenberg de Jong and Kruyt in 1929 (*2*, *12*). Coacervates are condensed droplets formed by liquid-liquid phase separation of macromolecules through multiple associative interactions, including electrostatics and hydrophobics (*13*). Coacervate droplets are membraneless and can incorporate these macromolecular building blocks by natural partitioning, leading to the sustained growth of the existing droplets. They can undergo repeated division via shape instabilities induced by chemical reaction (active division) or shearing (passive division) and can support prebiotically relevant nonenzymatic RNA copying (*14*–*16*). The hypothesis of coacervates as protocells has one key advantage over membrane-bound compartments: their ability to concentrate various molecules such as peptides, nucleotides, polymers, ionic surfactants, and fatty acids by simple partitioning where the transport of these molecules is controlled not by their size but by their affinity toward the coacervate matrix (*16*–*23*). However, recent studies have shown a notable drawback of this supposedly advantageous property of membraneless coacervate protocells. It has been observed that there is a rapid exchange of RNA molecules between coacervate protocells due to the absence of a membrane (*24*–*26*). An active exchange of RNAs between protocells would lead to identical complements of RNAs across all the protocells in a very short time. Such a process would decrease or eliminate competition between protocells under selection pressures. Therefore, a rapid and uncontrolled exchange of RNA between coacervate protocells would thwart the evolution of RNA-based life.

In addition to the above considerations, coacervate-based membraneless protocell models are limited by their tendency to fuse actively with neighbors, again promoting RNA exchange while leading to the macrophase separation of a thick layered fluid over a few hours, resulting in the loss of microcompartment structure. Stability against coalescence is essential to cellular life, as it allows cells to maintain their individuality and integrity during their life span. Modern cells achieve stability through the presence of a lipid membrane or a cell wall. Thus, stability can be achieved by building a membrane around an otherwise unstable complex fluid, inhibiting the fusion of droplets such as coacervates. We note that “stability” here actually corresponds to an out-of-equilibrium metastable state of arrested coalescence. Coacervate droplets have been stabilized against coalescence by the interfacial self-assembly of amphiphilic molecules, such as block copolymers, comb polymers, and phospholipid nanoparticles (*21*, *27*, *28*). However, the probability of occurrence of these specialized molecules in abundance in the prebiotic era seems very low due to their complex architecture. Moreover, membranes would obstruct the natural partitioning of charged macromolecules such as polyelectrolyte building blocks and RNA into the droplet and prevent droplet growth (*23*, *24*). Thus, while the rapid exchange of RNA across membraneless droplets, along with their coalescence, would lead to genetic homogeneity and obviate any selective advantage resulting from mutant ribozymes with superior functionality, the presence of a stabilizing membrane would impede droplet growth. Therefore, a prebiotically plausible coacervate stabilization mechanism that could allow droplet growth to occur while preventing genetic randomization is required.

Here, we adopt a minimalistic design to achieve compartmentalization of RNA in membraneless coacervate protocells that exhibit a size-dependent gradient in exchange rates of RNA between neighboring protocells while remaining stable against coalescence. We studied coacervates formed by poly(diallyldimethylammonium chloride) (PDDA), a positively charged polymer, and adenosine triphosphate (ATP), a nucleotide with four negative charges as model protocells that are highly unstable to coalescence (*29*). Upon shearing these coacervates in distilled water, we found that the protocells gained exceptional interfacial stability against fusion, lasting several months. We attribute this stability to a series of physicochemical changes at the droplet interface—a sudden discontinuity in ion concentration across the interface leading to a loss of interfacial counterions to bulk water, thus inducing electrostatic crosslinking between the charged macromolecules at the protocell interface (*30*). Despite being “membraneless” (in the sense of having no lipid or block copolymer membrane), these stable protocells exhibited controllable exchange of macromolecular cargo (proteins and RNA) across populations. We found that while small uncharged molecules and shorter RNA sequences of 6 to 8 nt exchange within a timescale of minutes, longer RNA sequences of 35 nt or higher remained compartmentalized for days, potentially allowing copies of catalytic RNA sequences to be passed down to daughter protocells without dilution in the environment or excessive exchange with other protocells (nt stands for the number of nucleotides in RNA). In addition, we found that this compartmentalization remained effective even at elevated temperatures, potentially assisting partial denaturation of RNA secondary structures necessary for prebiotic primer extension. Overall, our experiments show that low salinity freshwater—from sources such as rain, lakes, and melting of snow and ice—could have been a driver for the minimalistic stable compartmentalization of RNA before the advent of more complex, membrane-bound protocells.

## RESULTS

### Coacervates are inherently unstable and form “leaky” protocells

**Fig. 1.**
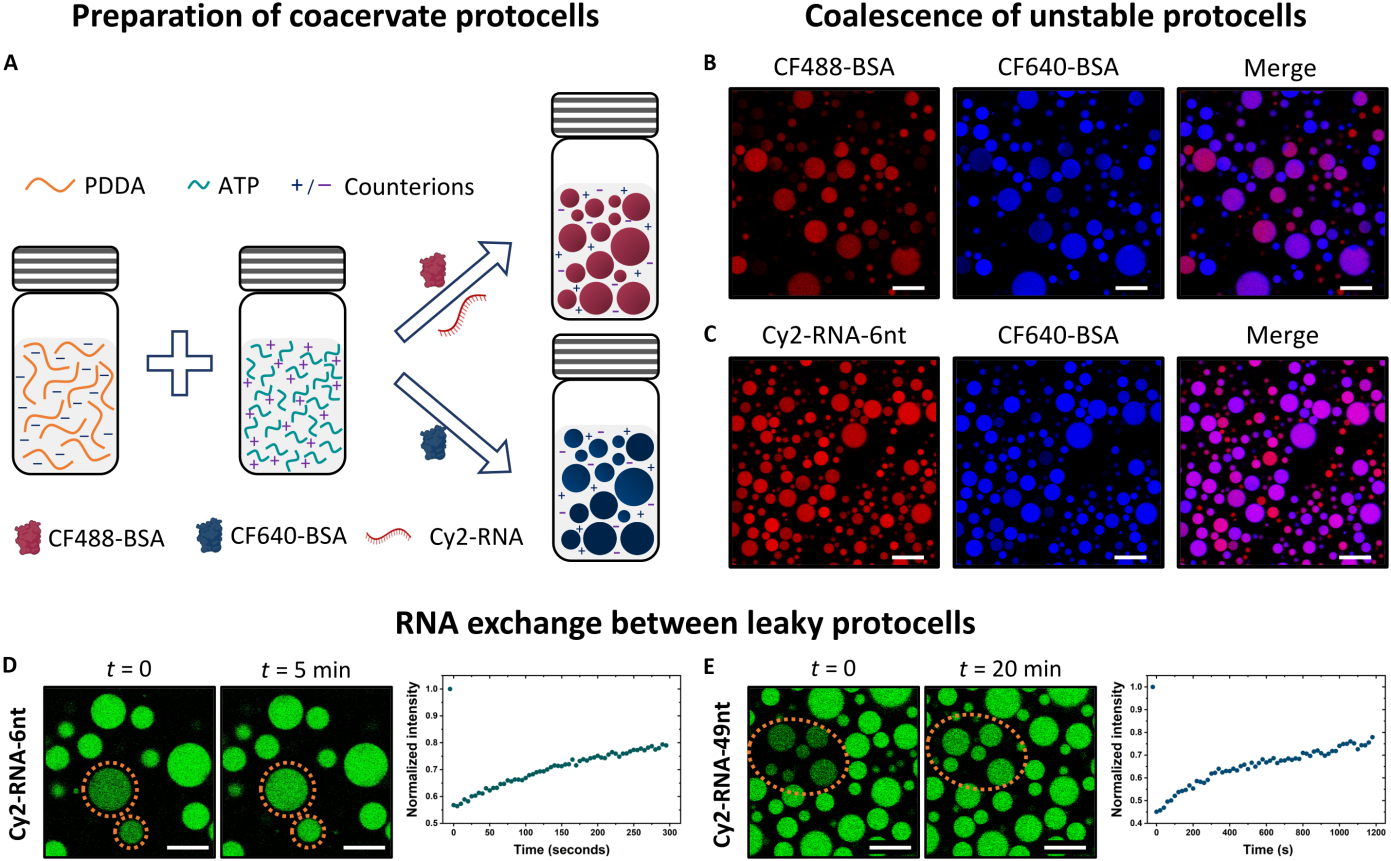
Rapid coalescence and RNA exchange between unstable protocells. (**A**) Schematic of preparation of coacervate model protocells. Aqueous solutions of PDDA (positively charged) and ATP (negatively charged) are mixed, along with fluorescently labeled protein or oligonucleotide molecules, to make fluorescently distinct protocell populations. (**B** and **C**) Confocal micrographs showing the coalescence of unstable protocells during mixing. Fluorescently distinct PDDA-ATP droplet populations containing fluorophore-labeled BSA or RNA were mixed, and the images were collected after 5 min. Coalescence is evident from the overlapping of red [CF488-BSA in (B) or Cy2-RNA-6nt in (C)] and blue (CF640-BSA) fluorescence in a majority of the droplets. Scale bars, 25 μm. (**D** and **E**) Confocal micrographs and the accompanying FRAP plot showing a fast (timescale of a few minutes) recovery of fluorescence from Cy2-labeled RNA of (D) 6 nt in length and (E) 49 nt in length in the photobleached droplets, suggesting a rapid exchange of RNA molecules between protocells. The fluorescence microscopy images are false colored for clarity. Scale bars, 25 μm.

Coacervate droplets were prepared in plastic centrifuge tubes by mixing aqueous solutions of PDDA and ATP (Fig. 1A for schematic and fig. S1 for structure). While PDDA is a synthetic polycation, PDDA-ATP is a well-studied model system and the findings in this study should apply to systems with more prebiotically plausible polycations, such as polypeptides with lysine or arginine-rich domains or low complexity polyamines such as spermine and spermidine. PDAA and ATP were mixed in a 1:1 stoichiometric charge ratio to a final concentration of 20 and 5 mM, respectively (pH = 8.0, mM stands for mmol/liter). The solution instantly turned turbid upon mixing, a characteristic of liquid-liquid phase separation where a polymer-rich phase (coacervate microdroplets) is formed, suspended in equilibrium in a polymer-lean phase that is usually rich in expelled counterions (supernatant) (*31*). Fluorescently conjugated protein molecules [bovine serum albumin (BSA): CF488-BSA and CF640-BSA] or RNA molecules (Cy2-labeled, 6 and 49 nt long; see table S1 for RNA sequences used) are added with ATP in the above mixing steps. Both BSA and RNA are negatively charged at pH 7 or higher and thus partition preferentially into the coacervate phase due to nonspecific charge-charge interactions (*32*, *33*). As expected, these droplets were unstable against coalescence. When two droplet populations containing distinct fluorescently labeled cargo molecules were mixed, the droplets coalesced within a few minutes, and this coalescence led to the mixing of cargo and a loss of individual identities of the droplets (Fig. 1, B and C, and movie S1). Moreover, fluorescence recovery after photobleaching (FRAP) showed that even when the droplets were spatially apart so that they could not fuse, an active exchange of fluorescently labeled RNA was happening between them, regardless of RNA length (Fig. 1, D and E). This rapid exchange of RNA (τ ≈ 1 min for 6 nt and τ ≈ 5 min for 49 nt RNA) in prebiotic scenarios would lead to homogenous distribution of genetic material across protocell populations over very short timescales (min) compared to the hours to days long timescales of prebiotic RNA replication and the growth and division of membrane-delimited model protocells (*34*–*36*).

### Stabilization of coacervate protocells against coalescence with distilled water

**Fig. 2.**
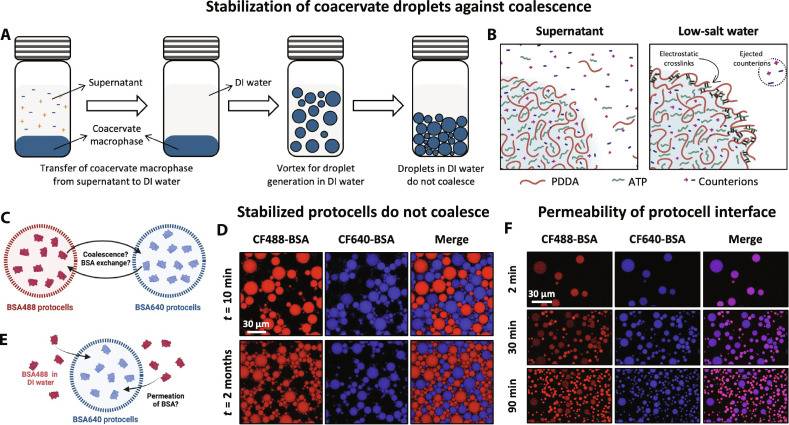
Stabilization of PDDA-ATP coacervate droplets in DI water. (**A**) Schematic of coacervate droplet stabilization achieved by transferring and shearing coacervate macrophase in DI water. (**B**) Schematic depicting the proposed mechanism for arrested coalescence. Droplet (blue hue) suspended in supernatant (i.e., equilibrium dilute phase, left) or low salt concentration water (DI water or rain/freshwater, right). (**C**) Illustration and (**D**) confocal micrographs of mixed droplet populations with two distinct fluorescently labeled BSA cargos showing stabilized droplets of PDDA-ATP coacervates neither coalesce nor exchange their protein cargo even after 2 months of stabilization. Images show individual channels (CF488-BSA as red and CF640-BSA as blue) and the composite image (merge). (**E**) Illustration and (**F**) confocal micrographs showing permeation of CF488-BSA (red) in stabilized droplets of PDDA-ATP coacervates preloaded with CF640-BSA as cargo (blue). The sample was vortexed before imaging for homogenous mixing of CF488-BSA, and the droplets were transferred to the well plate. Note that due to the slow sedimentation of smaller droplets, a larger number of them could be seen at longer times.

To stabilize coacervate model protocells against fusion, we transferred the coacervate macrophase (5 μl), extracted from the equilibrium supernatant phase, to distilled [deionized (DI)] water (1 ml), followed by vortex mixing (Fig. 2A). In a previous study, we proposed that this process imparts stability to the coacervate suspension by forming an electrostatically crosslinked layer of PDDA and ATP at the droplet-water interface (Fig. 2B) (*30*). When stabilized protocell populations containing different dye-labeled protein cargos were mixed, they did not fuse (Fig. 2, C and D). The sustained compartmentalization of BSA cargos in distinct droplet populations in these images also suggests that there was no exchange of proteinaceous cargos across droplet populations, and thus, these droplets are remarkably effective in storing their cargo. For as long as a month, these droplets not only resisted coarsening and macrophase separation but also preserved their protein cargos and, thus, conserved their identity (Fig. 2D).

To understand if the proposed interfacial crosslinking was “sealing off” the droplet interface, thereby inhibiting any potential diffusion of macromolecules across the interface, we added a small quantity of CF488-BSA (guest) to the water surrounding the stabilized CF640-BSA droplets (host) (Fig. 2E). We found that the droplet interface was highly permeable to these guest BSA molecules as they were able to diffuse across the interface to partition into the bulk of the host droplets (Fig. 2F). While this partitioning was low initially, it increased over time. Furthermore, we found that the partitioning increased faster in smaller droplets compared to that in larger ones. This observation can be explained by a higher surface-to-volume ratio of smaller droplets, where a concentration gradient-driven diffusion of guest molecules across the interface would rapidly enrich smaller droplets (see note S1). Overall, these observations confirm that while the proposed crosslinked interface of these stabilized droplets is quite porous to molecular diffusion, the protein molecules that are already partitioned inside these droplets remain compartmentalized and do not diffuse out. Next, we look at the stability of these model protocells against chemical changes.

### Externally added salt/acid destabilizes droplets to coalescence

**Fig. 3.**
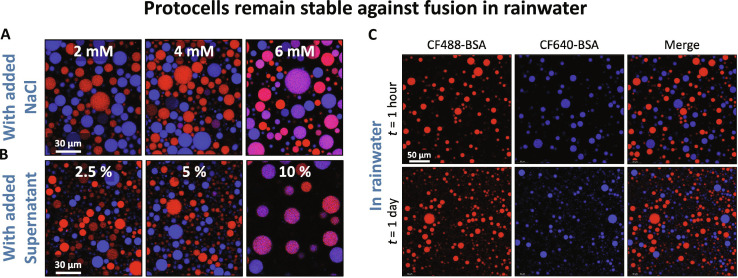
Protocell stability with externally added salt/supernatant and in rainwater. (**A** and **B**) Confocal micrographs (merged channels) showing coalescence of stabilized PDDA-ATP droplets containing CF488-BSA (red) and CF640-BSA (blue) as cargos with increasing (A) salt (in mM) or (B) supernatant concentration (by volume fraction) in the surrounding water. (**C**) Confocal micrographs showing the stability of PDDA-ATP droplets against fusion in rainwater after 1 hour (top row) and 1 day (bottom row). Droplets containing CF488-BSA and CF640-BSA were first prepared by shearing coacervate separately in rainwater and were then mixed.

Freshwater, for example, from rain or lakes, can contain dissolved counterions in trace amounts (i.e., in a few parts per million). Because the absence of counterions leads to droplet stability by proposed interfacial crosslinking, adding salt to the water surrounding the stable droplets could render them unstable. We tested this hypothesis by mixing the two fluorescently distinct droplet populations in NaCl solutions of varying ionic strength (Fig. 3A and fig. S2). At low salt concentration (*C*_s_ < 2 mM), the stabilized droplets remained stable and showed no sign of fusion. This salinity is much higher than what is commonly found in freshwater sources (*37*–*39*), suggesting that the droplets should remain stable against fusion in these freshwater sources. As the salt concentration was increased to 4 mM, a few of the droplets were found to fuse. At a salt concentration of 6 mM, there was a sudden jump in the extent of coalescence, suggesting that a threshold ion concentration is required for breaking the crosslinks between the interfacial macromolecules to allow for coalescence. This destabilizing salt concentration is of the same order as the counterion concentration in the supernatant of an unstable coacervate suspension (<12.7 mM; see note S2). Similarly, when we added the supernatant solution (which was in equilibrium with the unstable coacervate droplets) to the water surrounding the mixture of stable droplet populations, we found that the droplets remained relatively stable until 5% (by volume fraction) supernatant but became highly unstable against coalescence at a concentration of 10% supernatant (Fig. 3B and fig. S3). While 10% supernatant in DI water would correspond to roughly 1.3 mM NaCl (see note S2), a salt concentration at which the droplets seem to be relatively stable in Fig. 3A, the presence of PDDA and ATP molecules in the equilibrium supernatant may also contribute to destabilizing the droplet interface.

Because freshwater sources can have non-neutral pH, such as the acidic pH of rainwater, we performed experiments in acidic and alkaline water with pH ranging from 4 to 8 and found that the droplets prepared by transferring coacervates to these solutions did not coalesce (fig. S4). To further verify if rainwater could stabilize coacervate droplets against coalescence, we collected rainwater and performed experiments with it. The collected rainwater had a conductivity of 3.5 μS/cm, equivalent to 15.7 μM NaCl [although rainwater contains many other ions (*37*)] and a pH ca. 6. When the droplet populations prepared by transferring coacervate to this rainwater were mixed, they did not coalesce and remained stable for days (Fig. 3C). These experiments collectively suggest that the coacervate droplets can be stabilized against coalescence in low salt freshwater, such as rainwater, and this mechanism suggest a prebiotically plausible scenario for arresting the coalescence of coacervate based protocells.

### Proposed mechanism for arrested coalescence

Colloidal suspensions are usually stabilized (against aggregation or fusion) by electrostatic and/or steric repulsion. We measured the zeta potential of the unmodified droplets (i.e., those in their equilibrium supernatant) and of the stabilized droplets (i.e., those in DI water) to get an estimate of the surface charge on the droplets under the two different environments and found that the zeta potential value for droplets in DI water (+25 mV) was lower than that in the supernatant (+44 mV) (fig. S5). To minimize any artifacts arising from measurements in crowded suspensions, we performed measurements on suspensions diluted up to 100 times in their respective continuous phases (see Methods) and found that the zeta potentials of these suspensions plateaued at +26 mV for unmodified droplets and +25 mV for stabilized droplets. If charge-charge repulsion was responsible for arresting the coalescence of droplets in DI water, then this repulsion should also have been able to arrest the coalescence of unmodified droplets in the equilibrium supernatant having similar potential values. Because droplets coalesce readily in the supernatant, electrostatic repulsion is not responsible (or at least is not sufficient on its own) for arresting the coalescence of droplets in DI water. Thus, we conclude that the droplets are stabilized against coalescence primarily via steric repulsion.

Oppositely charged polyions attract each other, and in the absence of a sufficient concentration of salt/counterions that would otherwise help in screening their charges, this attraction would lead to the formation of solid aggregates (precipitates) or gels. This has been shown for the complexation of polystyrene sulfonate (PSS) with PDDA at different salt concentrations (*40*). We propose that when coacervates are transferred to DI water and sheared, the counterions near the droplet interface rapidly diffuse out from the droplet interface into bulk water. We detected this ejection of counterions in our conductivity measurements (see note S2). This loss of counterions would reduce the screening of charges on PDDA and ATP, allowing strong charge-charge associations (intrinsic ion-pairs) between these oppositely charged molecules (Fig. 2B and fig. S6). These strong associations, which we refer to as electrostatic crosslinks, would result in the formation of a thin elastic membrane at the surface of the droplets, thereby arresting coalescence.

Our primary hypothesis is that this mechanism arises from rapid complexation of the charged polymers of opposite sign, akin to gelation, under conditions in which the free more mobile counterions are depleted. This hypothesis is supported by previous observations from Luo *et al.* (*41*) who observed that one can form strong gels from polyelectrolytes by subjecting the as-prepared polyelectrolyte complexes to dialysis to slowly extract the mobile counterions from the complexes. To understand if our coacervate droplets crosslink only on the interface or if they become crosslinked throughout, giving rise to a percolated network (or a gel-like state), we performed FRAP measurements to monitor the diffusion of a dye-labeled, long-chain polyelectrolyte in the bulk of the droplets. We found that the diffusivity of the polyelectrolyte chains did not alter significantly upon the transfer of coacervate droplets into DI water, showing that the interior of the droplets remains in a liquid-like state (fig. S7). This conclusion is supported by previous rheological measurements where the viscous modulus was observed to be much higher than the elastic modulus of the bulk coacervate phase of both unmodified and stabilized droplets (*30*). This observation suggests the absence of percolation that might have been an alternate explanation of arrested coalescence and limited RNA exchange that we discuss later (*42*).

The above observation also implies that there should be enough salt in the bulk coacervate to maintain the liquid-like state even after the transfer of droplets into DI water. Quantitatively, 20 mM PDDA and 5 mM ATP would bring 20 mM Na^+^ and Cl^−^ ions with them, making the initial NaCl concentration 20 mM. Our conductivity measurements showed that the concentration of NaCl in the equilibrium supernatant after coacervate formation was approximately 12.7 mM. Given that ca. 11 μl of bulk coacervates was formed per milliliter of solution, the concentration of NaCl inside the bulk coacervate should be at least 670 mM. To form stabilized droplets, 5 μl of this bulk coacervate was suspended in 1 ml of DI water, after which the concentration of ejected Na^+^ and Cl^−^ ion in the water was found to be 0.36 mM. This measurement indicates that the concentration of NaCl inside the droplets is still at least 604 mM, i.e., 90% of the counterions that came in with the coacervate macrophase (670 mM) remained inside the droplets. These observations collectively suggest that the stability against coalescence of these coacervate droplets in DI water is achieved by steric repulsion due to the formation of a thin interfacial layer of electrostatically crosslinked PDDA and ATP. We think that this droplet skin also controls the flow of macromolecules in and out of the underlying droplet.

### Functional model protocells with enzymatic catalysis

**Fig. 4.**
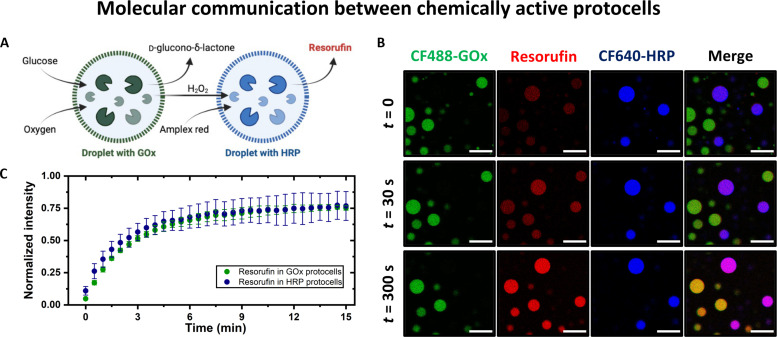
Droplet bioreactors demonstrating chemical communication. (**A**) Schematic of enzyme cascade in droplets and the corresponding droplet-to-droplet chemical communication. (**B**) Confocal micrographs showing the time evolution of resorufin fluorescence (red channel) in PDDA-ATP coacervate droplets with CF488-GOx (green channel) and CF640-HRP (blue channel) enzymes as cargo. Glucose and amplex red were introduced into the solution surrounding the droplets, and the resultant resorufin fluorescence first appeared in HRP droplets (merged channel color change from blue to purple to pink) and then in GOx (color change from green to yellow) droplets. Scale bars, 30 μm. A set of images from another independent trial of the same experiment are shown in fig. S7, where similar features were found. (**C**) A plot of time evolution of resorufin fluorescence intensity in stabilized droplets during enzyme catalysis. For each time point, normalized intensity corresponds to area fraction of HRP (or Gox) protocell covered by resorufin fluorescence, *n* = 4 images were analyzed, and error bars represent SDs.

Taking advantage of the durable protein compartmentalization capabilities of these stable model protocells, we prepared droplets containing enzyme molecules as cargo to explore their ability to act as chemically functional compartments (Fig. 4A). We chose an enzymatic cascade reaction enabled by glucose oxidase (GOx) and horseradish peroxidase (HRP), where β-d-glucose molecules are oxidized by GOx while producing hydrogen peroxide, H_2_O_2_, which is used by HRP to oxidize amplex red to resorufin (a fluorescently active molecule). Thus, the progress of this reaction can be tracked and quantified using fluorescence microscopy. We began by preparing two different stable droplet populations with CF488-GOx and CF640-HRP as cargos using our stabilization scheme. These droplet populations were then mixed along with amplex red, and the sample was transferred to an imaging chamber setup on the microscope. Lastly, glucose solution was added to this mix with gentle mixing, and a time-lapse imaging sequence was commenced. Immediately upon imaging, resorufin fluorescence was found in HRP droplets, signifying molecular communication between droplets, driven by the transport and exchange of substrate and product molecules through the semipermeable droplet interface (Fig. 4B and fig. S8). Because of the semipermeable nature of the droplet interface, the substrate molecules (glucose and amplex red) could diffuse into the droplets, thereby forming the products in the presence of respective enzymes, and these reaction products (hydrogen peroxide and resorufin) are exchanged between the droplets. Fluorescence from the resorufin channel was found to increase first in the HRP droplets (where it is produced) and then in the GOx droplets (where it reaches after diffusing out from HRP droplets), signifying its effective sequestration in coacervates (low background fluorescence) while exchanging actively between droplets (Fig. 4C). The resorufin fluorescence intensities in both the droplet populations become similar at long times and reach a plateau. These observations suggest that while substrate and product molecules can exchange via diffusion across droplets, possibly due to their small size and low specific interaction with coacervates, the enzyme molecules, owing to their electrostatic and possibly other interactions, remain compartmentalized in these droplets.

### Size-dependent RNA compartmentalization in stabilized droplets

**Fig. 5.**
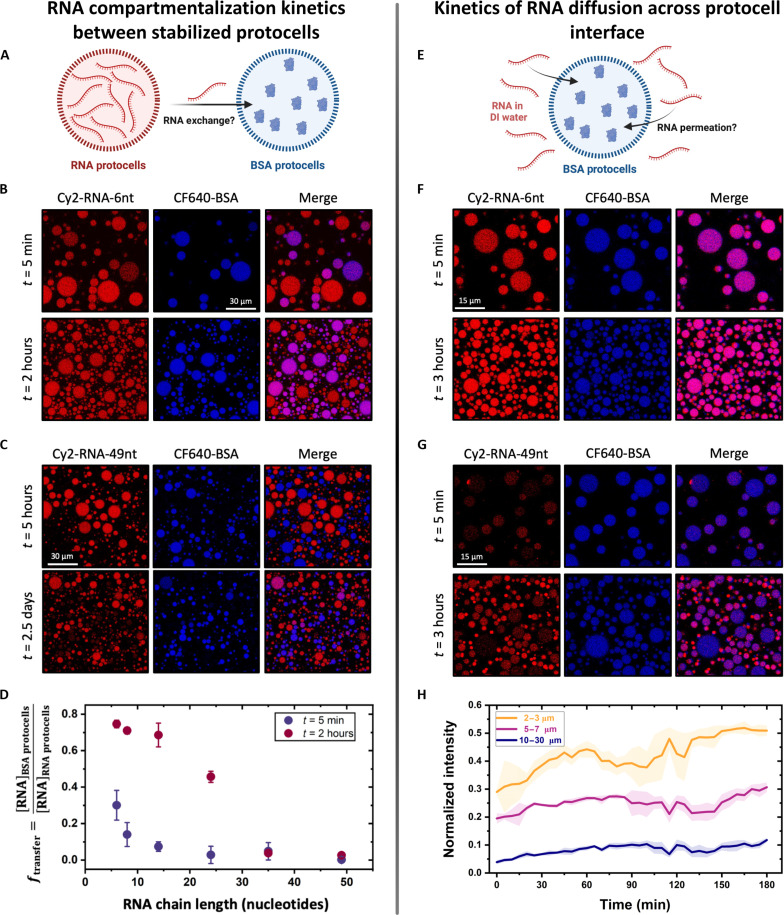
Size-dependent RNA compartmentalization and transfer in stabilized droplet. (**A**) Schematic of the experiment to detect RNA exchange in stabilized PDDA-ATP droplets using mixed droplet populations containing either Cy2-labeled RNA or CF640-labeled BSA. (**B**) Confocal micrographs showing transfer of short RNA chain, Cy2-RNA-6nt (red), from its own droplets to CF640-BSA droplets (blue) at 10 min and 2 hours after mixing. An increased number of smaller droplets could be seen due to prolonged sedimentation, while the larger droplets drifted out of the frame. (**C**) Confocal micrographs showing compartmentalization of Cy2-RNA-49nt (red) in its own droplet with no (5 hours) or some (2.5 days) transfer to CF640-BSA droplets (blue). (**D**) Plot showing the fraction of RNA transferred from RNA droplets to BSA droplets as a function of their chain length at 5 min and 2 hours. For each data point, *n* ≥ 6 samples were analyzed, and error bars represent SDs. (**E**) Schematic of the experiment to verify RNA transfer from surrounding solution into the stable BSA droplets across the “crosslinked” droplet interface. (**F**) Confocal micrographs showing short RNA oligos, Cy2-RNA-6nt (red), diffused and partitioned into BSA droplets (blue) immediately after mixing. (**G**) Confocal micrographs showing the inability of long RNA chain, Cy2-RNA-49nt (red), to diffuse into the BSA droplets immediately after mixing. Even at 3 hours after mixing, larger droplets remained devoid of the RNA oligos. (**H**) Plot showing the increase in area-averaged fluorescence intensity of Cy2-RNA-49nt as a function of time from droplets in three different size ranges. The smaller droplets accumulated more RNA than to the larger ones. The time axis in this plot is the imaging timestamp and does not include the sample preparation time of approximately 5 min. For each time point, *n* = 4 samples were analyzed, and error bars represent SDs.

Because the stabilized droplets did not exchange their protein cargos with each other, we proceeded to examine the compartmentalization of RNA cargos (Fig. 5A and see table S1 for sequences). To this end, we mixed two stabilized droplet populations in equal volumes: one containing a 6-nt-long RNA, Cy2-RNA-6nt, as its cargo (RNA droplets), and the other containing CF640-BSA as its cargo (BSA droplets). Because CF640-BSA molecules stay compartmentalized in droplets over a month, the BSA droplets served as a control to study any possible diffusion-driven exchange of RNA. When observed under a confocal microscope shortly after mixing (*t* ≈ 5 min), we found that Cy2 fluorescence was present in the BSA droplets as well, suggesting that RNA diffused out of their own droplets and diffused into the BSA droplets (Fig. 5B). This transfer increased over time as seen by the increase in Cy2 fluorescence in the BSA droplets over the course of 2 hours (Fig. 5B and fig. S9). In contrast, no CF640-BSA fluorescence was observed in the original RNA droplets (the original RNA droplets had high Cy2 fluorescence intensity and no observable CF640 fluorescence at *t* ≈ 0), showing that BSA molecules remain compartmentalized in BSA droplets and that this system of droplets was stable against droplet coarsening, as coalescence would lead to a distribution of BSA across all droplets over time, which was not observed.

When the same experiment was repeated with a longer chain length of 49 nt RNA (R49 or Cy2-RNA-49nt), we found no evidence of RNA exchange among droplets up to 5 hours after mixing (Fig. 5C). We could see an appreciable transfer of this long-chain RNA across droplets only after 2 days, suggesting that the rate of exchange of this RNA is very slow (Fig. 5C). This observation is in sharp contrast to the short RNA oligo tested, demonstrating effective spatial and temporal compartmentalization of a long RNA oligo in stabilized droplets. The very different results obtained with short and long RNAs suggested that the diffusion of RNA across the crosslinked interface of droplets might be chain length dependent. To test this hypothesis, we repeated these experiments with RNAs of varying sizes and found that the retention of RNA in stable droplets was indeed a function of their lengths. When imaged right after mixing (*t* ≈ 5 min), RNAs of length 6 nt, 8 nt, and, to a lesser extent, 14 nt were found in the BSA droplets, and their concentration in those droplets increased over time (Fig. 5D and fig. S10). Quantification of fluorescence intensity from these images revealed a noticeable decrease in the exchange of 8-nt RNA compared to the 6-nt RNA at short times (fig. S9). Furthermore, RNAs of length 24 nt appeared only in their own droplets initially, but they diffused into the BSA droplets to a visible extent in 2 hours (fig. S10). For RNAs of 35 and 49 nt in length, we did not see any “leakage” to BSA droplets for at least 6 hours, and even after 2 days, the extent of exchange was relatively low compared to shorter RNAs that exchange in minutes to hours (Fig. 5D and fig. S11).

Secondary structuring of RNA oligos due to intrachain base pairing can decrease their effective size (collapsed chain compared to a random coil), potentially affecting their exchange. This turned out to be a potential issue for R49 as it contained 20 random nucleotides at the end of the sequence, which led to the formation of multiple secondary structures (table S1, note S3, and fig. S12). To understand how secondary structures of RNA would affect their leakage, we studied the exchange of a different 49-nt RNA sequence that folds into a particular structure (named flexizyme), giving a predominant secondary structure that was smaller than the structures formed by the 49-nt sequence (fig. S12). We found that the exchange of flexizyme (Cy2-RNA-49nt-Flexi) was slightly faster than that of R49. For the initial period of 5 hours, we did not see any appreciable exchange of Cy2-labeled flexizyme across droplets, similar to what we found for R49 (fig. S13). The exchange became evident only after 2 days when flexizyme was present across a majority of the BSA droplets, quite in contrast to R49.

### Porosity of the interfacial layer controls RNA exchange between stabilized droplets

The hindered exchange of longer RNA oligos between stabilized droplets could be due to the following changes upon stabilization: either an increase in the viscosity of the coacervate matrix that would lead to slower diffusion of RNA oligos or the emergence of a physical barrier for diffusion of RNA across the droplet interface due to the proposed interfacial crosslinking or some combination of both. Previous measurements of the diffusion coefficient of polyelectrolyte chains inside droplets using FRAP have shown that the viscosity of the coacervate droplets does not change greatly upon stabilization (*30*). Moreover, when we tried to measure the diffusion coefficient of RNA oligos in unstable versus stabilized droplets using FRAP by partially bleaching the center of the droplets, we could not get a reliable photobleached image due to the very fast diffusion rates of RNA oligos. The diffusivities of RNA oligos were likely faster than the capture time of the camera used (0.2 s per frame for a 3-μm-wide circular bleached area in a 10-μm-diameter droplet). Therefore, decreased diffusion of RNA in the coacervate matrix was not responsible for slowing down the exchange of RNA oligos across droplets for larger RNAs.

To understand if the emergence of a physical barrier on droplet interface in DI water limits the exchange of RNA oligos, we looked at the rate of diffusion of RNA across the droplet interface by introducing stabilized droplets into a dilute solution of Cy2-labeled RNA and tracking change in its intensity in the droplets over time (Fig. 5E). Short RNA oligos of 6 nt in length were found to be present in the BSA droplets immediately after mixing, albeit with slightly different concentrations in different droplets, as evident from differences in fluorescence intensities from those droplets (Fig. 5F). Over time, this concentration increased and became uniform across droplets. In contrast, the uptake of 49-nt-long RNA oligos was much slower, as initially only a very low amount of RNA was present in the droplets (Fig. 5G). This amount increased steadily over time, and while smaller droplets concentrated a significant amount of RNA after 3 hours, it remained nearly absent in some of the larger droplets. As before, the diffusion-controlled transport of RNA into a droplet varied inversely with the droplet's size (Fig. 5H and note S1).

The difference in the timescale of uptake into the droplet between 6-nt RNA (less than 5 min) and 49-nt RNA (more than 3 hours) in stabilized droplets is much larger than in the unmodified droplets (~ minutes, as measured via FRAP; Fig. 1, D and E). Thus, it appears that the diffusion of RNA chains across the stabilized droplet interface is controlled by the porosity of the proposed interfacial crosslinking layer formed on the stabilized droplets in DI water. These pores restrict the diffusion of longer RNA oligos across the droplet interface while enabling the transport of the shorter RNAs. The critical size of the RNA that can be effectively compartmentalized inside the droplets thus depends on the porosity of the crosslinked interface. Various molecular parameters, such as polyelectrolyte chain length and nucleotide size, as well as system parameters, such as pH, ionic strength, and counterion valency, are expected to control the pore size.

### Compartmentalization remains effective even at elevated temperatures

**Fig. 6.**
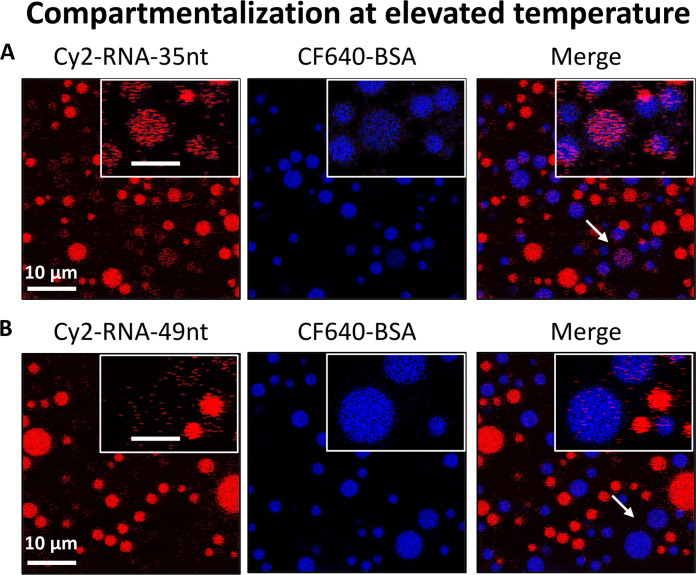
RNA compartmentalization remains effective at elevated temperatures. Confocal micrographs showing Cy2-RNA (red, left) and CF640-BSA (blue, center) fluorescence from mixed droplet populations after they were heated for 2 hours at 50°C. A low yet appreciable exchange of 35-nt RNA can be seen (**A**), while 49-nt RNA remained compartmentalized (**B**). Arrows indicate droplets that are magnified for clarity in the insets. Scale bars, 5 μm (inset). The brightness and contrast of the inset images have been enhanced for clarity, and the same processing has been applied to all the inset images to eliminate any bias.

Temperature and thermal gradients have been proposed to have played a crucial role in the evolution and dynamics of the prebiotic world, for example, by influencing the availability and reactivity of essential organic molecules (*43*, *44*). Thermal gradients or fluctuations might also have influenced RNA chemistry by assisting the denaturation (strand separation) of short duplexes (*3*). Thus, it is crucial to understand the stability of droplets at moderately elevated temperatures. We heated the mixed droplet populations of RNA and BSA to 40°C to see any sign of RNA or BSA exchange at this elevated temperature. This heating did not induce any exchange of Cy2-RNA-35nt or Cy2-RNA-49nt with surrounding BSA droplets, and the compartmentalization was maintained even after 2 hours (fig. S14). After heating the same sample again at a further elevated temperature of 50°C for 2 hours, we did see a slight exchange of 35-nt RNA between droplets (Fig. 6A). For the longer 49-nt RNA, this exchange was largely absent (Fig. 6B). Thus, these stabilized droplets can keep RNA oligos compartmentalized at elevated temperatures, potentially enabling oligonucleotide replication chemistry during out-of-equilibrium thermal cycles (*45*).

## DISCUSSION

Could stabilized coacervate droplets function as primordial protocells during the origin of life? The finding that coacervate droplets can be stabilized against coarsening by transferring them into distilled water revives the possibility that life may have emerged in compartments that formed spontaneously by the phase separation of charged polymers. The stabilization technique we used requires only that coacervate droplets be exposed to distilled water to form stabilizing interfacial crosslinks. The fact that rainwater is essentially distilled water, nearly free of ions, and was common on the primordial Earth, suggests that coacervate droplets could have been stabilized by rainfall or by transport in low salt freshwater environments. We suggest that Darwinian evolution may have been possible in coacervate droplet protocells in which the compartmentalization of long RNA oligonucleotides is maintained as a result of the absence of droplet fusion and the restricted exchange of RNA between droplets. While the effective spatiotemporal compartmentalization of long oligonucleotides is a prerequisite for Darwinian evolution, the fast exchange of short oligos (<10 nt) could be either beneficial, e.g., by providing raw material for RNA replication, or detrimental, e.g., by interfering with RNA replication via nonspecific interactions. In the stabilized coacervate droplets that we have examined, the exchange of RNAs depends on both chain length (short versus long) and shape (random coil versus secondary structure). To allow for the long-term maintenance of the genetic identity of individual droplets, a further reduction in RNA exchange is likely to be required. We suggest that this might be accomplished by varying the extent of interfacial crosslinking by using different polyelectrolytes having strongly or weakly charged functional groups than those used here.

Differences in the rates of diffusion of RNAs into coacervate droplets and the differences in the rates of exchange of RNAs between droplets could allow for the emergence of complex multidroplet systems using diffusion-limited reaction pathways (*46*). Alternatively, the formation of internal subcompartments in the form of multiphase coacervates, where two or more immiscible membraneless coacervates coexist in a hierarchical morphology, may allow for the spatiotemporal organization of reaction chemistry (*47*, *48*). When we used coacervates formed by a different polyanion, carboxymethyl dextran (CMD), with PDDA, we did see the formation of RNA compartments within stabilized PDDA-CMD coacervate droplets (fig. S15), suggesting that hierarchical complexity can be further elaborated with different polyelectrolyte systems.

The enzymatic cascade reaction that we studied using a dual coacervate droplet population was chosen to demonstrate the potential for chemical communication between protocells. In principle, chemical communication could also be mediated by RNA chemistry relevant to the origin of life. For example, protocells could use self-splicing RNAs or ribozyme-assisted substrate cleavage reactions to signal neighboring protocells by sending short RNA strands as their cleaved products over short timescales (*28*, *49*–*51*). The exchanged shorter strands could then modulate ribozyme activity or take part in ligation chemistry in the receiving protocell. In the context of ribozyme chemistry, it is noteworthy that these reactions require Mg^2+^ ions for ribozyme function, and many studies have shown that Mg^2+^ ions partition preferentially into the coacervate phase (*33*).

The ability of stabilized protocells to spontaneously recruit oligonucleotides and peptides from the surrounding water through its porous interface could have allowed for droplet growth. While we have not yet studied the growth and division of stabilized coacervate droplets, we expect them to grow following the addition of building blocks (polyelectrolytes and nucleotides), given that we have demonstrated the uptake of these charged macromolecules. Moreover, while the crosslinking of the interface may suggest that the droplets would resist shear-driven fission, the interface does deform to counter external pressure, as previously described (*30*). This deformation may allow droplets to divide, although likely at higher shear rates than for unmodified droplets. Clearly, the ability of stabilized coacervate droplets to undergo cycles of growth and division requires further study. On the primordial Earth, membraneless compartments that could grow and divide, and with the appropriate internal RNA chemistry to allow for RNA replication, could potentially propagate and evolve.

Overall, the stabilized coacervate protocells we have introduced here address two of the foremost challenges facing coacervates as protocells: fusion and exchange. Hence, this protocell model opens possibilities for studies of the origin of life and raises many exciting questions such as: How can the size selectivity of exchange be manipulated by the choice of coacervates polyelectrolytes? How is droplet stability affected by divalent ions, and how is their partitioning affected by the stabilization process? And how could changes in RNA exchange due to changes in environmental conditions, such as salinity and rain, have affected the overall process of evolution? Future studies on RNA chemistry, such as nonenzymatic primer extension inside stabilized coacervate protocells, could elucidate these phenomena in more detail.

## METHODS

### Materials

PDDA (8.5 kDa) was purchased from Polysciences. ATP disodium salt, NaCl, NaOH, HCl (2 mol/liter), d-(+)-glucose, and polyvinylpyrrolidone (PVP) (360 kDa) were purchased from TCI Chemicals. Reactive fluorescent dyes [*N*-hydroxysuccinimide (NHS) ester–functionalized CF488A and CF640R] and fluorescently labeled BSA (CF488A tagged: CF488-BSA and CF640R tagged: CF640-BSA) were purchased from Biotium. GOx (G2133), HRP (P8375), ammonium acetate, EDTA, formamide, and isopropanol were purchased from MilliporeSigma. Amplex red (A12222), dimethyl sulfoxide (DMSO), and triethylamine trihydrofluoride (TEA; 3HF) were purchased from Thermo Fisher Scientific. All reagents were used without further purification unless otherwise noted. Distilled water (DI water; 18 megohm cm) was obtained using the Milli-Q system from MilliporeSigma.

### Oligo synthesis

Cy2-labeled RNA oligonucleotides (see table S1 for sequences) were synthesized in-house on a K&A H-6 DNA/RNA synthesizer. The reagents and phosphoramidites were purchased from ChemGenes (Wilmington, MA) and Glen Research (Sterling, VA). After the synthesis, the RNA was cleaved from the solid support by incubation with 1.5 ml of 1:1 by volume mixture of 28% aqueous ammonium hydroxide and 40% aqueous methylamine for 15 min at room temperature. After cleavage, the nucleobases were deprotected in the same solution for 15 min at 65°C. Following a brief cool down, the solutions were evaporated in a speed-vac concentrator for 2 hours and lyophilized for 16 hours. The 2′-*O*-TBMDS protecting groups were removed from the lyophilized RNA by redissolving it in 100 μl of anhydrous DMSO and 125 μl of TEA.3HF and heating it at 65°C for 2.5 hours. The fully deprotected RNA was then diluted with 22.5 μl of ammonium acetate (5 mol/liter) and 1 ml of isopropanol and allowed to precipitate for 20 min on dry ice. The RNA was pelleted, washed with 80% ethanol, redissolved in a solution of 5 mM EDTA in 99% formamide (by volume fraction), and purified by denaturing urea–polyacrylamide gel electrophoresis. After the desired RNA band was cut out of the gel, it was crushed and soaked in a solution of 5 mM sodium acetate (pH 5.5) and 2 mM EDTA (pH 8.0) (overall unadjusted pH = 7.0) for 16 hours. The gel pieces were filtered away with a 5-μm syringe filter, and the RNA was desalted using the Waters (Milford, MA) C18 Sep-Pak cartridge.

### Dye labeling of enzymes

Protein labeling used an amine-reactive fluorophore to covalently attach the label. Lyophilized enzyme (2 mg; GOx or HRP) was added to 1 ml of sodium bicarbonate buffer (pH 8.0). To this mixture, *N*-hydroxysuccinimidyl ester–functionalized fluorophores were added in 1:1 stoichiometry (1.25 μl of NHS-CF488A for GOx or 5 μl of NHS-CF640R for HRP) from a 10 mM stock in DMSO. The solutions were vortex-mixed and kept in the dark for 2 hours at room temperature for the reaction to occur. After the reaction, the mixture was transferred to 10-kDa molecular weight cutoff ultracentrifuge filter tubes, which were then centrifuged at 12,000*g* until the solution was reduced to 50 μl. Unreacted dye molecules passed through the filter, while dye-conjugated enzymes remained in the tube. The labeled fraction was washed with buffer two times and then once with DI water to ensure the complete removal of unreacted labels. The labeled enzymes were made up to 1 ml with DI water to ensure the initial concentration of 2 mg/mL. These stocks were stored at 4°C.

### Preparation of coacervates and stabilization in DI water

Coacervates were prepared by adding a stock solution of ATP to DI water, followed by PDDA, to a final concentration of 20 mM PDDA (monomer basis, 20 mM positive charge) and 5 mM ATP (20 mM negative charge). Charge balance conditions have been found to allow maximum yield of coacervate from phase separation. The pH of the final solution was maintained at 8.0 using NaOH to allow complete deprotonation of ATP. For preparing coacervates with cargo, dye-labeled BSA (or RNA, GOx, and HRP) molecules were added before the addition of PDDA to a final concentration of 10 nM. A polyelectrolyte mixture (1 ml) yielded around 11 μl of coacervate macrophase, suggesting that the final concentration of dye-labeled molecules in the coacervates was roughly 1 μM. To prepare a mixture of droplet populations, equal volumes of coacervate droplet suspensions from two desired samples were pipetted into a vial and mixed on a vortex shaker for 30 s before transferring to a 96-well plate for imaging.

To prepare stabilized protocells, coacervate macrophase was collected by centrifuging the above droplet-supernatant suspension at 1000*g* for 5 min, followed by pipetting out 5 μl of macrophase and resuspending it in 1 ml of DI water. To prepare mixtures of stable coacervate droplet populations, equal volumes of droplet-water suspensions from the two samples were pipetted into a vial and mixed on a vortex shaker for 30 s and then transferred to a 96-well plate for imaging. In all the experiments, mixing was done using a vortex mixer, and samples were pipetted out shortly after to avoid sedimentation-related changes in droplet concentration.

### Zeta potential

Zeta potential measurements of coacervate droplets (in equilibrium supernatant or DI water) were performed on an electrophoretic light scattering instrument (Litesizer 500, Anton Paar) equipped with a 658-nm laser using the proprietary omega cuvettes from Anton Paar. To prepare samples at different dilutions, the continuous phase of any given droplet suspension was isolated by centrifuging the suspension at 5000*g* for 5 min and collecting the top, droplet-free phase, followed by its filtration through a 0.22-μm filter. The dilutions were prepared by mixing the droplet suspensions in their respective isolated continuous phase, preserving the background ion concentration of continuous phase.

### Enzyme cascade experiments

A 50 μl each of stabilized GOx and HRP coacervate droplet suspensions was pipetted into a plastic centrifuge vial. To this mixture, 1 μl of amplex red from a 10 μM stock in DMSO was added, and the suspension was mixed and transferred to a 96-well plate placed on the microscope. Lastly, to initiate the cascade reaction, 50 μl of 1 mM glucose was pipetted into the well, followed by gentle mixing using pipette aspiration, and imaging.

### Confocal microscopy, FRAP experiments, and partition coefficients

Coacervate droplet suspensions were imaged in coverglass bottom 96-well plates (Cellvis P96-1.5H-N) that were precoated with PVP to prevent the wetting of coacervate droplets on the glass bottom. The coating was achieved by washing in sequence with NaOH (1 M), HCl (1 M), DI water, and 2% (by mass) PVP solution in water (for at least 2 hours). Images were acquired on a laser scanning confocal microscope (SP8, Leica) equipped with three excitation lasers (ex) at approximately 1% or less laser intensity, used for imaging CF488A or Cy2 (488-nm ex), resorufin (560-nm ex), and CF640R (640-nm ex), with proper excitation/emission filter sets. The LAS X software from Leica was used to control the microscope and acquire images using a 20× or 63× magnification oil-immersion objective lens, as needed.

FRAP experiments were done on the same setup by photobleaching a small region of interest in one or more coacervate droplets using 100% laser intensity for a few seconds, followed by usual image acquisition at 1% or less laser intensity at short intervals over a few minutes to acquire fluorescence recovery kinetics. Partition coefficients were measured using images acquired on the confocal microscope at 1% laser intensity and a photonmultiplier tube detector.

### Diffusion across porous protocell interface

The stabilized CF640-BSA coacervate droplet suspension (100 μl) was pipetted into a 96-well plate. To this well, 1 μl of either CF488-BSA from a 15 μM (1 mg/ml) stock solution or Cy2-RNA (of required nucleotide length) from a 1 μM stock solution was added, followed by gentle mixing using pipette aspiration and imaging.

### Protocell stability with externally added ions (salt, supernatant, acid, or base) and in rainwater

For experiments with external salt addition, stock solutions of NaCl were prepared by dissolving NaCl powder in DI water. For experiments with external supernatant addition, the supernatant solution was isolated by centrifuging initial coacervate-supernatant suspension (not stabilized or BSA added). A total of 50 μl each of stabilized CF488-BSA and CF640-BSA coacervate droplet suspensions were pipetted in a plastic centrifuge vial. To this mixture, 10 μl of NaCl stock solution or supernatant was added in the required amount, and the suspension was again mixed and transferred to a 96-well plate for imaging. For experiments in acidic/alkaline water, hydrochloric acid or sodium hydroxide was added to DI water in required amounts and the pH was adjusted using a pH meter (from Mettler Toledo). For experiments with rainwater, rainwater was collected outside our laboratory in the city of Houston during a recent rain in March 2024.

## Supplementary Materials

### This PDF file includes:

Notes S1 to S3 Figs. S1 to S15 Table S1 Legend for movie S1 References
