# Supplementary material for: Did the exposure of coacervate droplets to rain make them the first stable protocells?
Source: Sci Adv. 2024 Aug 21;10(34):eadn9657. doi: 10.1126/sciadv.adn9657 (PMC11338219; doi:10.1126/sciadv.adn9657)
Supplement: Supplementary file 1 — Notes S1 to S3 Figs. S1 to S15 Table S1 Legend for movie S1 References [file sciadv.adn9657_sm.pdf]

Science Advances
AAAS

Supplementary Materials for

Did the exposure of coacervate droplets to rain make them the first stable protocells?

Aman Agrawal *et al.*

Corresponding author: Aman Agrawal, amanagrawal@uchicago.edu; Matthew V. Tirrell, mtirrell@uchicago.edu; Alamgir Karim, akarim3@central.uh.edu; Jack W. Szostak, jwszostak@uchicago.edu

Sci. Adv. **10**, eadn9657 (2024)
DOI: 10.1126/sciadv.adn9657

The PDF file includes:

Notes S1 to S3
Figs. S1 to S15
Table S1
Legend for movie S1
References

Other Supplementary Material for this manuscript includes the following:

Movie S1

Note 1: Diffusion across droplet interface

When dye-labeled macromolecules, such as CF488-BSA or Cy2-RNA, are introduced into the solution surrounding the coacervate droplets, they partition into the protocells by diffusing across the protocell interface. It is evident from the microscopy images that these macromolecules strongly partition into the coacervate matrix. Given this high partitioning, the flux, j, of these molecules into the protocells can be modeled as a one-way transport at initial times. The number of molecules crossing the interface of a protocell with a radius r in a Δt time interval can be written as

$$N_{in} = j(4\pi r^2)(\Delta t)\ .$$

The concentration of these molecules inside the protocells of volume V_{in} can then be written as

$$C_{in} = \frac{N_{in}}{V_{in}} = \frac{j(4\pi r^2)(\Delta t)}{\frac{4}{3}\pi r^3} = \frac{3j\Delta t}{r}\ .$$

Thus, the concentration of partitioned macromolecules inside the protocells at any given time is inversely proportional to their radius, explaining the higher concentration of the guest molecules in the smaller protocells than the larger ones at initial times.

Note 2: NaCl concentration using conductivity measurements

To estimate the amount of counterion ejection from the droplet interface, we measured the conductivity of the water surrounding the stabilized droplets (originally deionized water, but not anymore due to the ejected counterions) and found it to be (45.2 $\pm$ 6.0) µS/cm. Using a calibration curve shown below on the left, we found that this conductivity corresponded to a salt concentration of (0.36 $\pm$ 0.036) mM NaCl (mM: mmol/L).

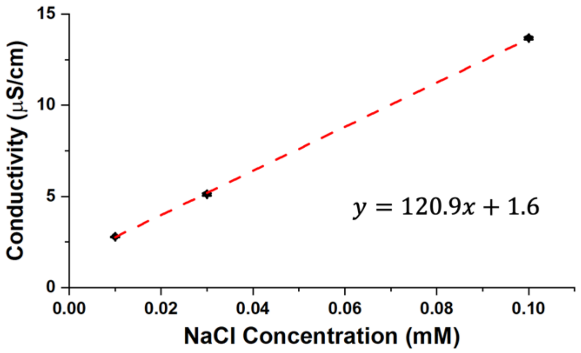

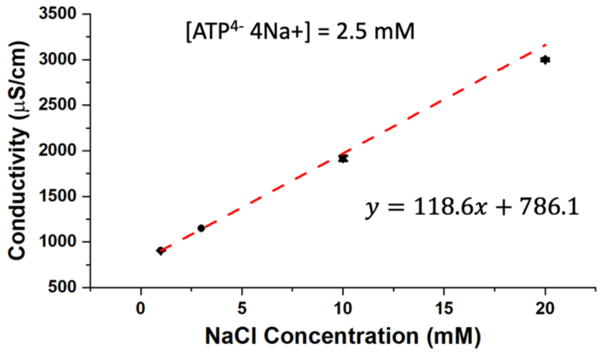

The conductivity of the original equilibrium supernatant was found to be (2295.6 $\pm$ 57.2) µS/cm, measured after centrifuging down the droplets to leave a clear supernatant, which was further filtered using a 0.22 µm filter. The supernatant was found to contain around 2.5 mM of adenosine triphosphate (ATP), which certainly contributed to its conductivity. Using a calibration curve shown above on right, we found that the NaCl concentration in the supernatant is nearly 12.7 mM. However, since there will be some poly(diallyldimethylammonium chloride) (PDDA) in the

supernatant in equilibrium with ATP (the concentration of which was not able to be measured due to experimental limitations), it will contribute to the conductivity of the supernatant. Thus, the actual salt concentration in the supernatant will be lower than 12.7 mM. Note that the fitted calibration curve in the plot on the right does not pass through the origin. This is due to the presence of 2.5 mM ATP sodium salt in the standard solutions, the conductivity of which at no added NaCl was found to be 731.2 ± 15.4 µS/cm, close to the intercept value of the calibration curve.

Below is a summary of the salt amount in the two phases at different stages:
Total NaCl in 1 mL original (unmodified) coacervate suspension: 20 mM x 1 mL = 20 µmol **(A)**
1 mL of coacervate suspension results in ca. 11 µL of coacervate macrophase. Thus,
NaCl in dilute phase: 12.7 mM x 989 µL = 12.6 µmol = 63% of A
NaCl in coacervate macrophase: 670 mM x 11 µL = 7.4 µmol = 37% of A
Now when 5 µL of coacervate is transferred to 1 mL DI water:
Total NaCl introduced into the system: 670 mM x 5 µL = 3.35 µmol **(B)**
NaCl ejected into DI water: 0.36 mM x 1 mL = 0.36 µmol = 10.7% of B
Thus, NaCl remained in stable coacervate droplets ~89.3% of B

Note 3: Secondary structuring of RNA sequences used

To understand the possibility of secondary structures formed by R49 due to the presence of random nucleotides at the 3' end of the sequence that may base pair intra-chain, we performed polyacrylamide gel electrophoresis (PAGE) of this sequence under native (non-denaturing) conditions. The experiments were performed in the absence of any divalent ions, as we did not use any divalent ions in our experiments. It is known that secondary structure(s) of RNA sequence form folded, compact structures that run faster on a native gel due to their compact size (*52*). We found that the R49 formed a smear, rather than a tight band, in the gel suggesting a range of secondary structures that ran on the gel at different speeds. Compared to R49, Flexizyme ran faster and in a tight band, suggesting most of the molecules had a compact secondary structure, that is smaller than R49.

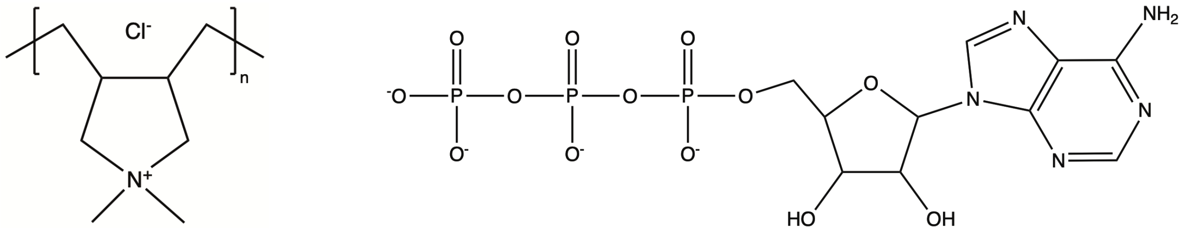

Figure S1: Chemical structures of polydiallyldimethylammonium chloride (PDDA, left) and adenosine triphosphate (ATP, right). PDDA has a chain length (n) of ca. 53 monomers. ATP molecules have Na^+ as the counterion (not shown).

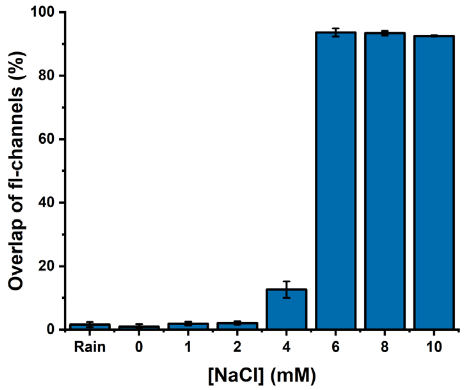

Figure S2: Plots showing the percentage of droplets containing both CF488-BSA and CF640-BSA when the two distinct droplet populations were stabilized and mixed in solutions of different salt concentrations.

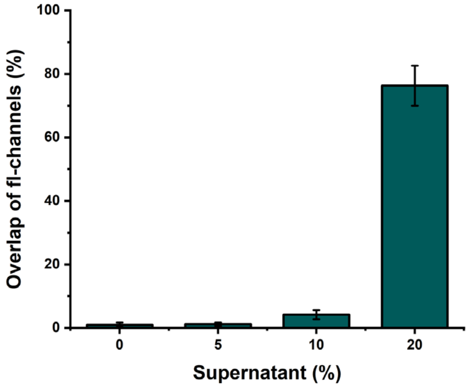

Figure S3: Plots showing the percentage of droplets containing both CF488-BSA and CF640-BSA when the two distinct droplet populations were stabilized and mixed in solutions of different supernatant fractions.

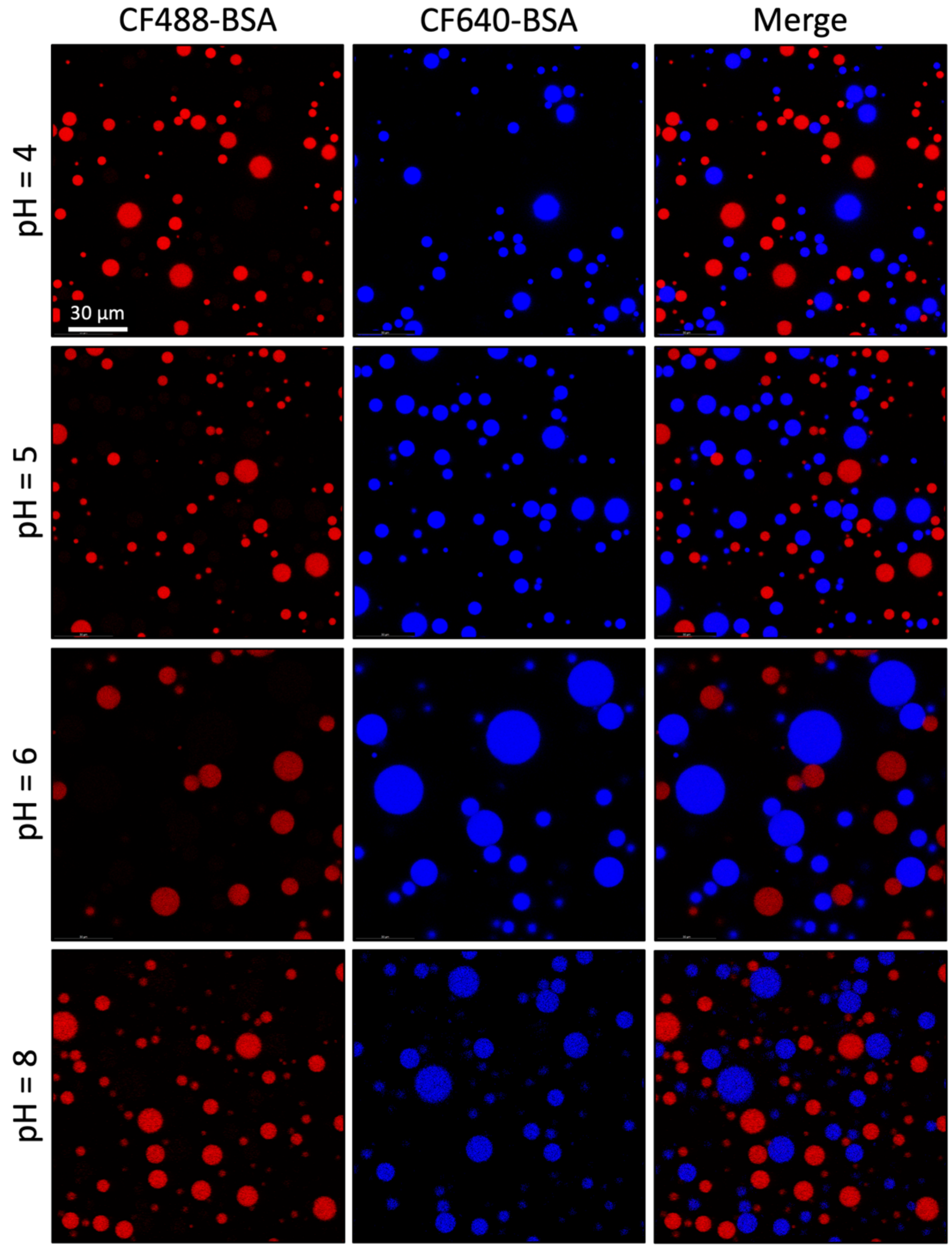

Figure S4: Confocal micrographs showing the stability of PDDA-ATP droplets in low-salt water of varying pH. Droplets containing CF488-BSA and CF640-BSA were first sheared separately in acidic water and were then mixed using a vortex mixer. The acidic water was prepared by adding HCl or NaOH to DI water at different concentrations. Images were taken 30 min after mixing.

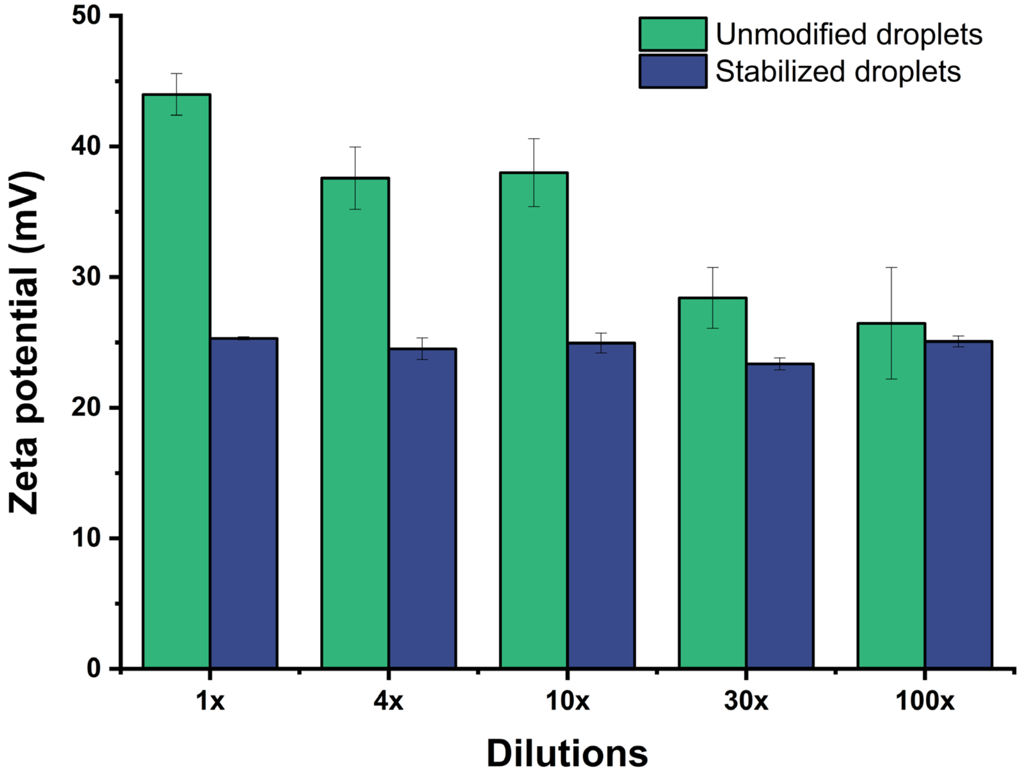

Figure S5: Measured zeta potential values for unmodified droplets in equilibrium supernatant and stabilized droplets in DI water at different dilutions. 1x denotes as-prepared suspensions. A 10x dilution, for e.g., corresponds to a sample where 40 μL of 1x as-prepared suspension was added to 360 μL of corresponding continuous phase.

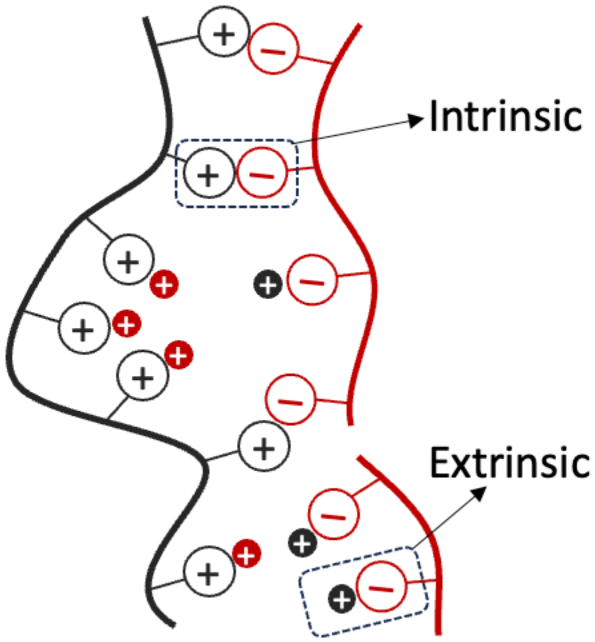

Figure S6: Schematic of different interactions in polyelectrolyte complexes (53). These interactions are broadly of two types: intrinsic and extrinsic ion pairs. Polyanion-polycation pairs are intrinsic ion pairs while polyelectrolyte-counterion pairs are extrinsic ion pairs. An electrostatically crosslinked layer refers to a state where the interfacial layer is dominated by intrinsic ion pairs.

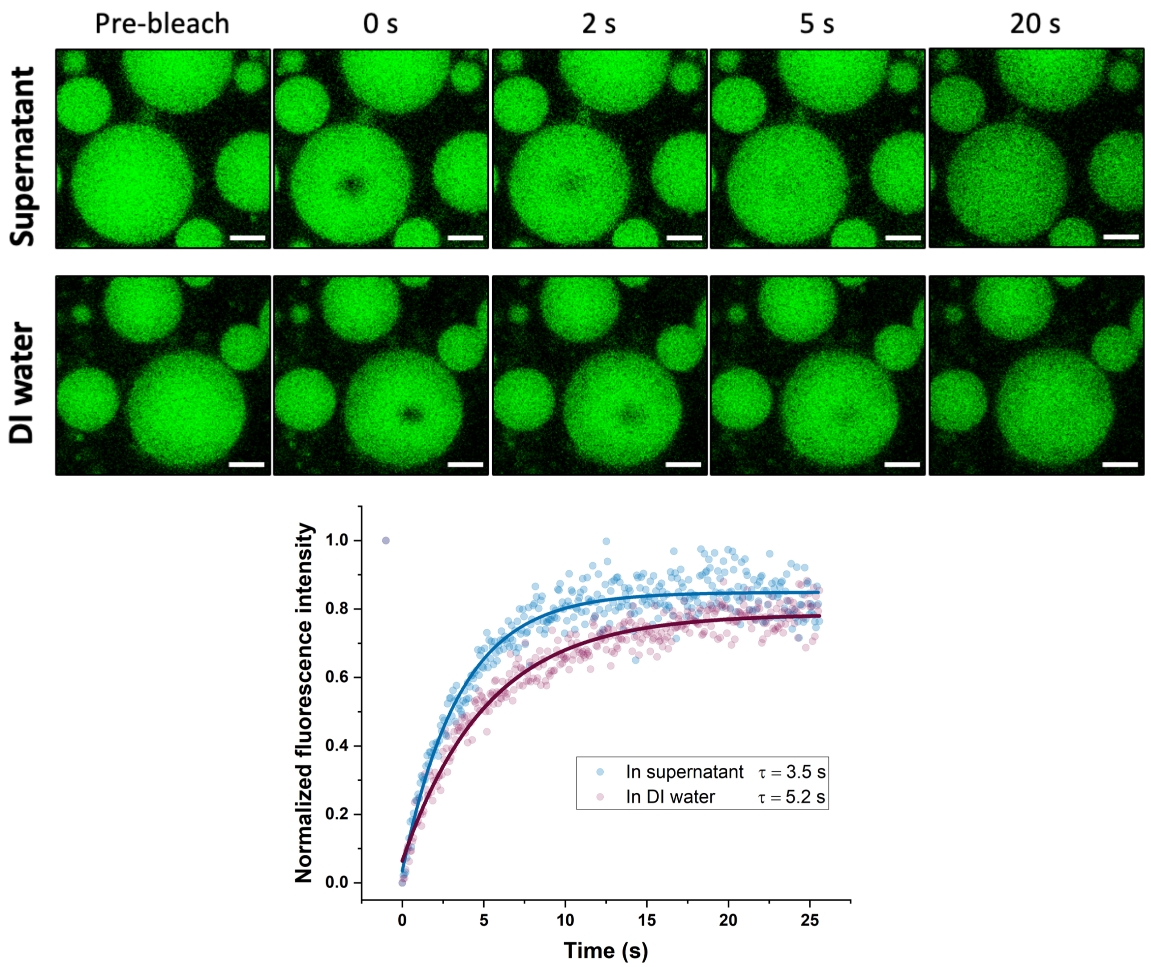

Figure S7: (Upper panel) Time series of confocal microscopy images of coacervate droplets in the supernatant (top) and DI water (bottom) before and after photobleaching. The fluorescence signal is from FITC labeled carboxymethyl dextran (ca. 900 monomers long) and the dark spot in the center of the droplet at t = 0 s is the photobleached region. Scale bar: 10 µm. (Lower panel) The recovery in fluorescence intensity from the photobleached region. 'τ' is the time constant of recovery from exponential fits as shown in solid curves.

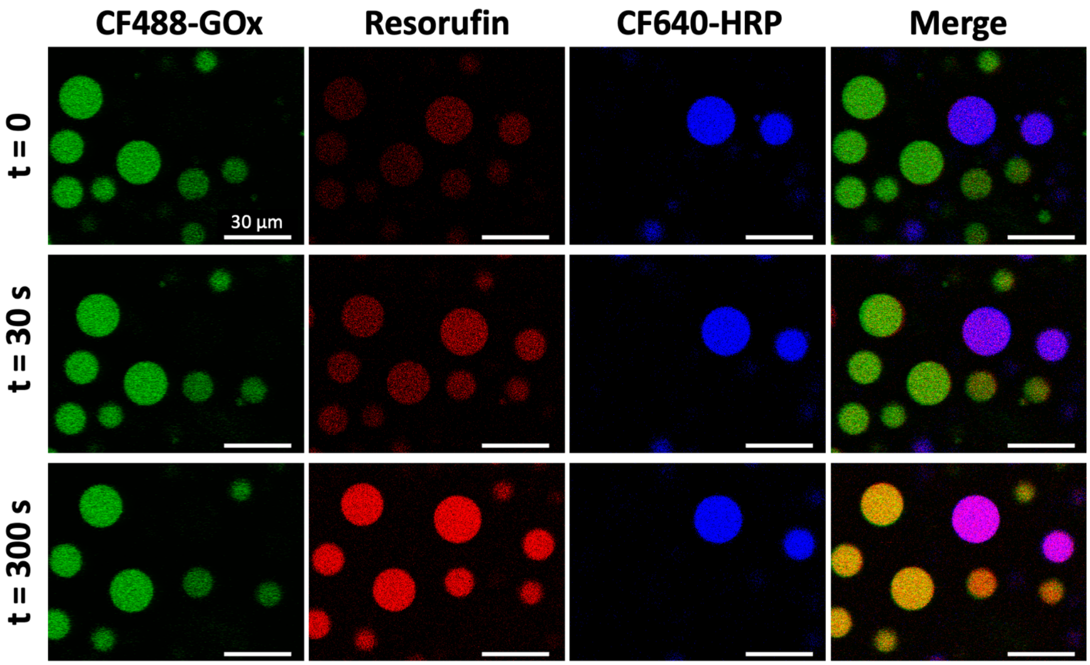

Figure S8: Fluorescence micrographs in a different trial showing the time evolution of Resorufin fluorescence (red channel) in PDDA-ATP coacervate protocells with CF488-GOx (green channel) and CF640-HRP (blue channel) enzymes as cargo. The formation of Resorufin in the HRP protocells is apparent with visibly higher intensity of Resorufin fluorescence in the HRP protocells at short times (t = 0 s and 30 s).

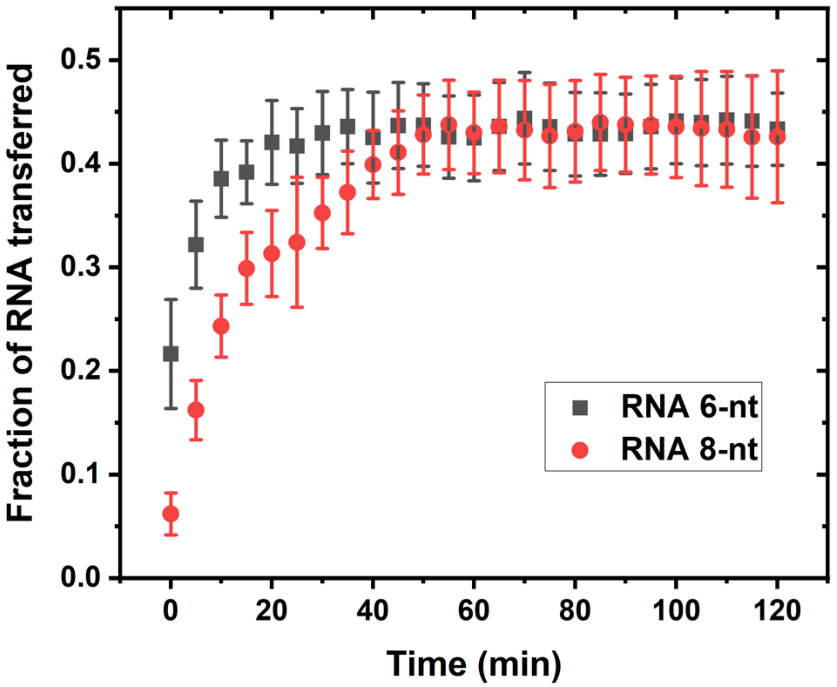

Figure S9: Fraction of RNA transferred from RNA protocells to BSA protocells over time for 6-nt and 8-nt long chains. The exchange of RNA of 6-nt length is quite rapid, as evidenced by the significant transfer fraction at t = 0 (when the first image was acquired). Mixing the samples and transferring them to the sample holders for imaging takes ~ 5 min. For each time point, n = 8 samples were analyzed, and error bars represent standard deviations.

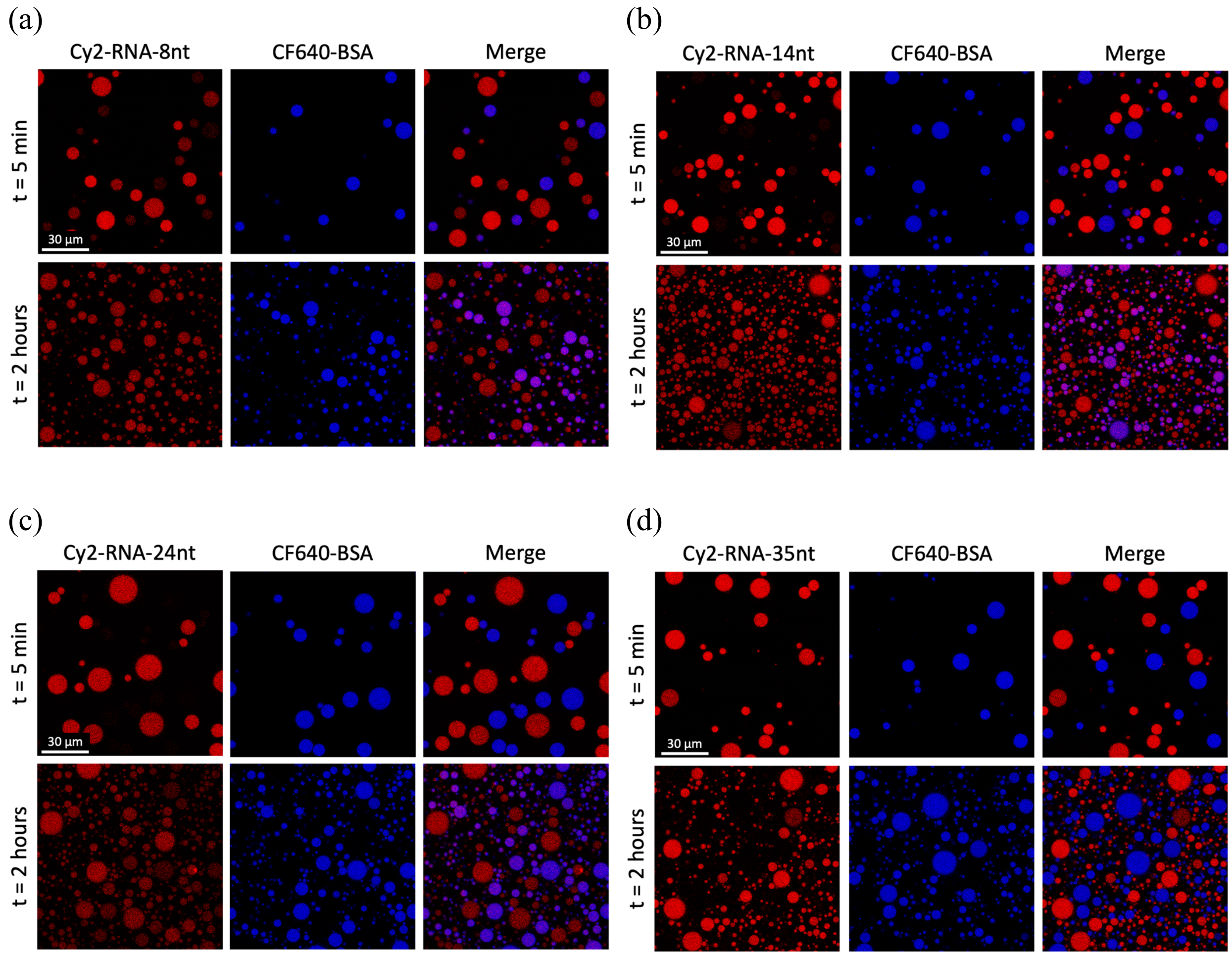

Figure S10: Confocal images showing changes in the distribution of RNAs of varying chain lengths of (a) 8-nt, (b) 14-nt, (c) 24-nt, and (d) 35-nt across protocells after 2 h.

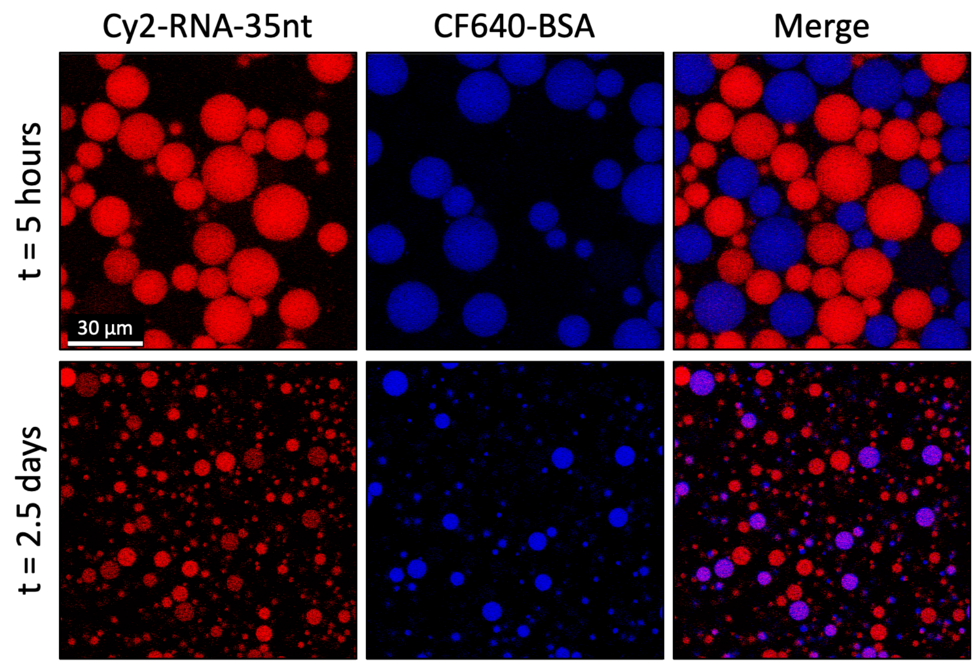

Figure S11: Confocal images showing changes in the distribution of Cy2-RNA-35nt across coacervate droplets between 5 h (top) and 2.5 d (bottom).

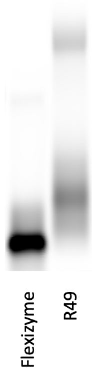

Figure S12: Fluorescence image of polyacrylamide gel showing size distributions of the Flexizyme and R49 sequences used in this study. ~1 µM of 5'-Cy2-labeled RNA was analyzed by polyacrylamide gel electrophoresis (PAGE) under native (non-denaturing) conditions in the absence of any divalent ions.

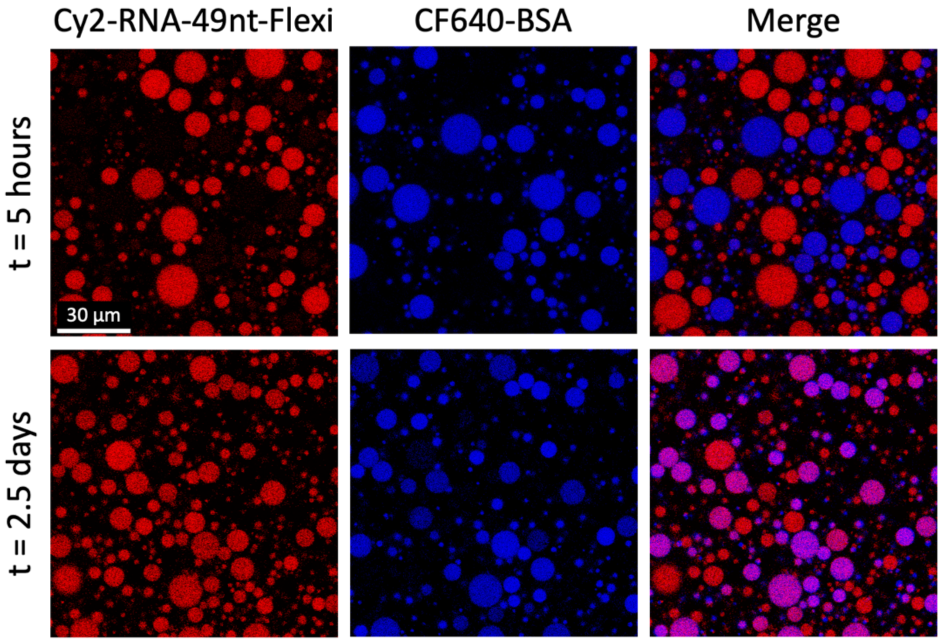

Figure S13: Confocal images showing changes in the distribution of Cy2-RNA-49nt-Flexi across coacervate droplets between 5 h (top) and 2.5 d (bottom).

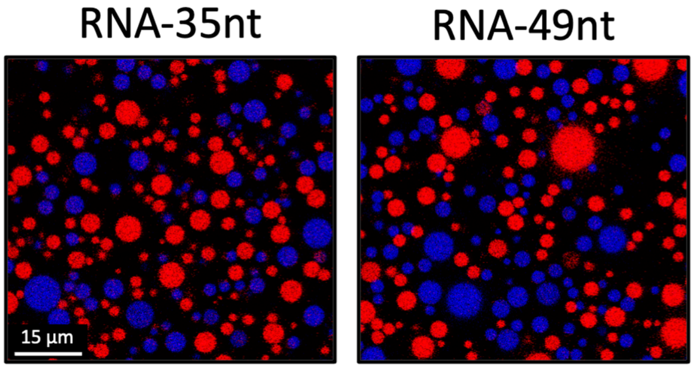

Figure S14: Confocal images showing BSA-640 droplets (blue) and Cy2-RNA-35nt (red, left) or Cy2-RNA-49nt (red, right) after 2 h of heating in an oven at an elevated temperature of 40 ºC.

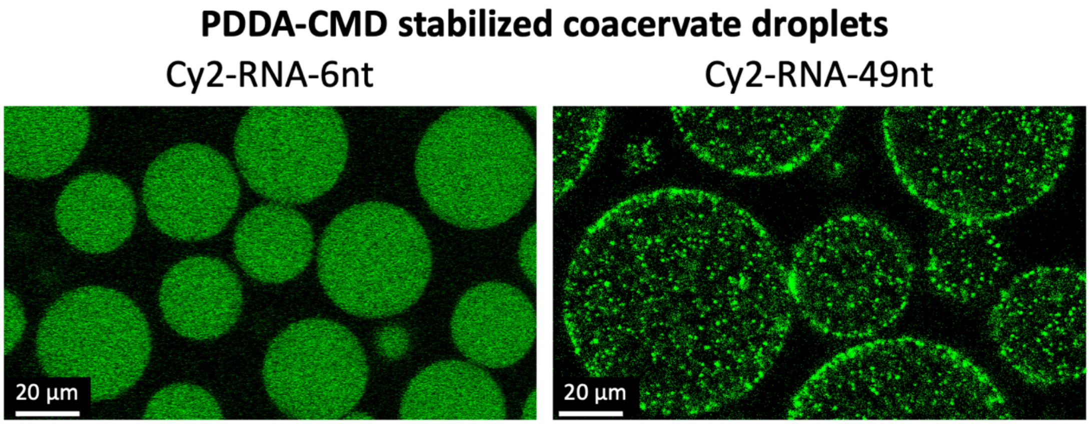

Figure S15: Confocal images showing stabilized coacervate protocells made by mixing PDDA and carboxymethyl dextran (CMD) containing a homogeneously distributed Cy2-RNA-6nt (left) or phase-separated Cy2-RNA-49nt (right).

Table S1: Nucleotide sequence of RNA used.

S. No.	Sequence	Name	Length (nt)
1	5'-cy2-GGGACA	RNA-6nt	6
2	5'-cy2-GGACCGCA	RNA-8nt	8
3	5'-cy2-GGAAGUUGUUAUCA	RNA-14nt	14
4	5'-cy2-CGCAUCCUCACGCAAGUGGGUACA	RNA-24nt	24
5	5'-cy2-GUUCAGAGUUCUACAGUCCGAC GAUCAAANNNNNN	RNA-35nt	35
6	5'-cy2-GUUCAGAGUUCUACAGUCCGACGAUC AAANNNNNNNNNNNNNNNNNNNN	RNA-49nt or R49	49
7	5'-cy2-GGACCGCAAGGUUCCGCAUCCUCAC GCAAGUGGGUACAUGGCGUUAGCU	RNA-49nt-Flexi or Flexizyme	49

Video S1: Time-lapse showing coalescence of unmodified PDDA-ATP coacervate droplets (i.e. in equilibrium supernatant) using brightfield microscopy. Encircled regions highlight fusion events. Time is in seconds, as shown in the top-right corner. Scale bar: 50 µm, as shown on the bottom-left corner.

REFERENCES AND NOTES

1. W. Snyder, S. W. Fox, A model for the origin of stable protocells in a primitive alkaline ocean. *Biosystems* **7**, 222–229 (1975).

2. A. I. Oparin, *The Origin of Life on the Earth* (Oliver & Boyd, Edinburgh & London, 1957), pp. 18–495.

3. J. W. Szostak, D. P. Bartel, P. L. Luisi, Synthesizing life. *Nature* **409**, 387–390 (2001).

4. P.-A. Monnard, P. Walde, Current ideas about prebiological compartmentalization. *Life* **5**, 1239–1263 (2015).

5. G. F. Joyce, RNA evolution and the origins of life. *Nature* **338**, 217–224 (1989).

6. W. Gilbert, Origin of life: The RNA world. *Nature* **319**, 618–618 (1986).

7. I. A. Chen, P. Walde, From self-assembled vesicles to protocells. *Cold Spring Harb. Perspect. Biol.* **2**, a002170 (2010).

8. T. F. Zhu, J. W. Szostak, Coupled growth and division of model protocell membranes. *J. Am. Chem. Soc.* **131**, 5705–5713 (2009).

9. S. S. Mansy, J. P. Schrum, M. Krishnamurthy, S. Tobé, D. A. Treco, J. W. Szostak, Template-directed synthesis of a genetic polymer in a model protocell. *Nature* **454**, 122–125 (2008).

10. S. S. Mansy, Membrane transport in primitive cells. *Cold Spring Harb. Perspect. Biol.* **2**, a002188 (2010).

11. G. Cooper, K. Adams, *The Cell: A Molecular Approach* (Oxford Univ. Press, 2023).

12. H. G. Bungenberg de Jong, H. R. Kruyt, Coacervation (partial miscibility in colloid systems), *Proc. Kon. Ned. Akad.* **32**, 849–856 (1929).

13. E. Gomes, J. Shorter, The molecular language of membraneless organelles. *J. Biol. Chem.* **294**, 7115–7127 (2019).

14. W. K. Spoelstra, E. O. van der Sluis, M. Dogterom, L. Reese, Nonspherical coacervate shapes in an enzyme-driven active system. *Langmuir* **36**, 1956–1964 (2020).

15. C. Donau, F. Späth, M. Sosson, B. A. K. Kriebisch, F. Schnitter, M. Tena-Solsona, H. S. Kang, E. Salibi, M. Sattler, H. Mutschler, J. Boekhoven, Active coacervate droplets as a model for membraneless organelles and protocells. *Nat. Commun.* **11**, 5167 (2020).

16. M. Matsuo, K. Kurihara, Proliferating coacervate droplets as the missing link between chemistry and biology in the origins of life. *Nat. Commun.* **12**, 5487 (2021).

17. D. Priftis, M. Tirrell, Phase behaviour and complex coacervation of aqueous polypeptide solutions. *Soft Matter* **8**, 9396–9405 (2012).

18. M. Zhao, S. A. Eghtesadi, M. B. Dawadi, C. Wang, S. Huang, A. E. Seymore, B. D. Vogt, D. A. Modarelli, T. Liu, N. S. Zacharia, Partitioning of small molecules in hydrogen-bonding complex coacervates of poly(acrylic acid) and poly(ethylene glycol) or pluronic block copolymer. *Macromolecules* **50**, 3818–3830 (2017).

19. P. M. McCall, S. Srivastava, S. L. Perry, D. R. Kovar, M. L. Gardel, M. V. Tirrell, Partitioning and enhanced self-assembly of actin in polypeptide coacervates. *Biophys. J.* **114**, 1636–1645 (2018).

20. M. Abbas, W. P. Lipiński, K. K. Nakashima, W. T. S. Huck, E. Spruijt, A short peptide synthon for liquid–liquid phase separation. *Nat. Chem.* **13**, 1046–1054 (2021).

21. S. Gao, S. Srivastava, Comb polyelectrolytes stabilize complex coacervate microdroplet dispersions. *ACS Macro Lett.* **11**, 902–909 (2022).

22. K. K. Nakashima, M. H. I. van Haren, A. A. M. André, I. Robu, E. Spruijt, Active coacervate droplets are protocells that grow and resist Ostwald ripening. *Nat. Commun.* **12**, 3819 (2021).

23. H. Jing, Y. Lin, H. Chang, Q. Bai, D. Liang, Mass transport in coacervate-based protocell coated with fatty acid under nonequilibrium conditions. *Langmuir* **35**, 5587–5593 (2019).

24. T. Z. Jia, C. Hentrich, J. W. Szostak, Rapid RNA exchange in aqueous two-phase system and coacervate droplets. *Orig. Life Evol. Biosph.* **44**, 1–12 (2014).

25. W. M. Aumiller, Jr., F. Pir Cakmak, B. W. Davis, C. D. Keating, RNA-based coacervates as a model for membraneless organelles: Formation, properties, and interfacial liposome assembly. *Langmuir* **32**, 10042–10053 (2016).

26. H. M. Fares, A. E. Marras, J. M. Ting, M. V. Tirrell, C. D. Keating, Impact of wet-dry cycling on the phase behavior and compartmentalization properties of complex coacervates. *Nat. Commun.* **11**, 5423 (2020).

27. A. F. Mason, B. C. Buddingh', D. S. Williams, J. C. M. van Hest, Hierarchical self-assembly of a copolymer-stabilized coacervate protocell. *J. Am. Chem. Soc.* **139**, 17309–17312 (2017).

28. D. C. Dewey, C. A. Strulson, D. N. Cacace, P. C. Bevilacqua, C. D. Keating, Bioreactor droplets from liposome-stabilized all-aqueous emulsions. *Nat. Commun.* **5**, 4670 (2014).

29. D. S. Williams, S. Koga, C. R. C. Hak, A. Majrekar, A. J. Patil, A. W. Perriman, S. Mann, Polymer/nucleotide droplets as bio-inspired functional micro-compartments. *Soft Matter* **8**, 6004–6014 (2012).

30. A. Agrawal, J. F. Douglas, M. Tirrell, A. Karim, Manipulation of coacervate droplets with an electric field. *Proc. Natl. Acad. Sci. U.S.A.* **119**, e2203483119 (2022).

31. L. Li, S. Srivastava, M. Andreev, A. B. Marciel, J. J. de Pablo, M. V. Tirrell, Phase behavior and salt partitioning in polyelectrolyte complex coacervates. *Macromolecules* **51**, 2988–2995 (2018).

32. K. A. Black, D. Priftis, S. L. Perry, J. Yip, W. Y. Byun, M. Tirrell, Protein encapsulation via polypeptide complex coacervation. *ACS Macro Lett.* **3**, 1088–1091 (2014).

33. E. A. Frankel, P. C. Bevilacqua, C. D. Keating, Polyamine/nucleotide coacervates provide strong compartmentalization of Mg2+, nucleotides, and RNA. *Langmuir* **32**, 2041–2049 (2016).

34. J. W. Szostak, The eightfold path to non-enzymatic RNA replication. *J. Syst. Chem.* **3**, 2 (2012).

35. K. Adamala, J. W. Szostak, Nonenzymatic template-directed RNA synthesis inside model protocells. *Science* **342**, 1098–1100 (2013).

36. R. R. Poudyal, R. M. Guth-Metzler, A. J. Veenis, E. A. Frankel, C. D. Keating, P. C. Bevilacqua, Template-directed RNA polymerization and enhanced ribozyme catalysis inside membraneless compartments formed by coacervates. *Nat. Commun.* **10**, 490 (2019).

37. D. Carroll, *Rainwater as a Chemical Agent of Geologic Processes: A Review* (U.S. Geological Survey, 1962).

38. H. A. Dugan, L. A. Rock, A. D. Kendall, R. J. Mooney, Tributary chloride loading into Lake Michigan. *Limnol. Oceanogr. Lett.* **8**, 83–92 (2023).

39. V. N. Nijampurkar, M. M. Sarin, D. K. Rao, Chemical composition of snow and ice from Chhota Shigri glacier, Central Himalaya. *J. Hydrol.* **151**, 19–34 (1993).

40. Y. Liu, B. Momani, H. H. Winter, S. L. Perry, Rheological characterization of liquid-to-solid transitions in bulk polyelectrolyte complexes. *Soft Matter* **13**, 7332–7340 (2017).

41. F. Luo, T. L. Sun, T. Nakajima, T. Kurokawa, Y. Zhao, K. Sato, A. B. Ihsan, X. Li, H. Guo, J. P. Gong, Oppositely charged polyelectrolytes form tough, self-healing, and rebuildable hydrogels. *Adv. Mater.* **27**, 2722–2727 (2015).

42. G. M. Wadsworth, W. J. Zahurancik, X. Zeng, P. Pullara, L. B. Lai, V. Sidharthan, R. V. Pappu, V. Gopalan, P. R. Banerjee, RNAs undergo phase transitions with lower critical solution temperatures. *Nat. Chem.* **15**, 1693–1704 (2023).

43. A. W. J. Muller, D. Schulze-Makuch, Thermal energy and the origin of life. *Orig. Life Evol. Biosph.* **36**, 177–189 (2006).

44. R. Attal, L. Schwartz, Thermally driven fission of protocells. *Biophys. J.* **120**, 3937–3959 (2021).

45. M. Todisco, J. W. Szostak, Hybridization kinetics of out-of-equilibrium mixtures of short RNA oligonucleotides. *Nucleic Acids Res.* **50**, 9647–9662 (2022).

46. A. Leathers, M. Walczak, R. A. Brady, A. al Samad, J. Kotar, M. J. Booth, P. Cicuta, L. di Michele, Reaction–diffusion patterning of DNA-based artificial cells. *J. Am. Chem. Soc.* **144**, 17468–17476 (2022).

47. G. A. Mountain, C. D. Keating, Formation of multiphase complex coacervates and partitioning of biomolecules within them. *Biomacromolecules* **21**, 630–640 (2020).

48. C. Donau, F. Späth, M. Stasi, A. M. Bergmann, J. Boekhoven, Phase transitions in chemically fueled, multiphase complex coacervate droplets. *Angew. Chem. Int. Ed.* **61**, e202211905 (2022).

49. K. Kruger, P. J. Grabowski, A. J. Zaug, J. Sands, D. E. Gottschling, T. R. Cech, Self-splicing RNA: Autoexcision and autocyclization of the ribosomal RNA intervening sequence of tetrahymena. *Cell* **31**, 147–157 (1982).

50. G. A. Prody, J. T. Bakos, J. M. Buzayan, I. R. Schneider, G. Bruening, Autolytic processing of dimeric plant virus satellite RNA. *Science* **231**, 1577–1580 (1986).

51. C. J. Hutchins, P. D. Rathjen, A. C. Forster, R. H. Symons, Self-cleavage of plus and minus RNA transcripts of avocado sunblotch viroid. *Nucleic Acids Res.* **14**, 3627–3640 (1986).

52. S. A. Woodson, E. Koculi, in *Methods in Enzymology* (Academic Press, 2009), vol. 469, pp. 189–208.

53. Y. Zhang, P. Batys, J. T. O’Neal, F. Li, M. Sammalkorpi, J. L. Lutkenhaus, Molecular origin of the glass transition in polyelectrolyte assemblies. *ACS Cent. Sci.* **4**, 638–644 (2018).
